# Tree Age Effects on Fine Root Biomass and Morphology over Chronosequences of *Fagus sylvatica*, *Quercus robur* and *Alnus glutinosa* Stands

**DOI:** 10.1371/journal.pone.0148668

**Published:** 2016-02-09

**Authors:** Andrzej M. Jagodzinski, Jędrzej Ziółkowski, Aleksandra Warnkowska, Hubert Prais

**Affiliations:** 1 Polish Academy of Sciences, Institute of Dendrology, Parkowa 5, PL-62-035 Kórnik, Poland; 2 Poznań University of Life Sciences, Faculty of Forestry, Department of Game Management and Forest Protection, Wojska Polskiego 71c, PL-60-625 Poznań, Poland; University of Saskatchewan, CANADA

## Abstract

There are few data on fine root biomass and morphology change in relation to stand age. Based on chronosequences for beech (9–140 years old), oak (11–140 years) and alder (4–76 years old) we aimed to examine how stand age affects fine root biomass and morphology. Soil cores from depths of 0–15 cm and 16–30 cm were used for the study. In contrast to previously published studies that suggested that maximum fine root biomass is reached at the canopy closure stage of stand development, we found almost linear increases of fine root biomass over stand age within the chronosequences. We did not observe any fine root biomass peak in the canopy closure stage. However, we found statistically significant increases of mean fine root biomass for the average individual tree in each chronosequence. Mean fine root biomass (0–30 cm) differed significantly among tree species chronosequences studied and was 4.32 Mg ha^-1^, 3.71 Mg ha^-1^ and 1.53 Mg ha^-1^, for beech, oak and alder stands, respectively. The highest fine root length, surface area, volume and number of fine root tips (0–30 cm soil depth), expressed on a stand area basis, occurred in beech stands, with medium values for oak stands and the lowest for alder stands. In the alder chronosequence all these values increased with stand age, in the beech chronosequence they decreased and in the oak chronosequence they increased until ca. 50 year old stands and then reached steady-state. Our study has proved statistically significant negative relationships between stand age and specific root length (SRL) in 0–30 cm soil depth for beech and oak chronosequences. Mean SRLs for each chronosequence were not significantly different among species for either soil depth studied. The results of this study indicate high fine root plasticity. Although only limited datasets are currently available, these data have provided valuable insight into fine root biomass and morphology of beech, oak and alder stands.

## Introduction

Although roots play a key role in tree access to soil resources (water and nutrient uptake) and mechanical stability, they are still a poorly understood component of forest ecosystems. Roots must be both extensive and sufficiently dynamic to meet the needs of the aboveground parts of trees which change noticeably with stand age. Fine roots, traditionally defined as all roots ≤ 2 mm in diameter [1], are especially important for tree growth and stand development; even though they are only a minor fraction of total tree biomass (less than 5%), they can use up to 30–50% of annual gross primary production (GPP), e.g. [2–3]. Moreover, they are considered highly dynamic and a functionally significant component of forest carbon and nutrient cycling and accumulation in the soil, due to their relatively fast decomposition compared to aboveground tree tissues (e.g. needles/leaves, branches) [4–6].

The majority of biomass studies published so far have focused on the stand structure and aboveground biomass responses to stand age. There are comparatively few data on how fine root biomass and morphology change in relation to forest stand age, even though stand-age-related variation in fine root biomass and morphological traits are of key importance to understand multifunctional, ecophysiological changes during stand development, e.g. [7–18].

Generally, fine root biomass (FRB) increases to a peak at canopy closure, after which it gradually declines in maturing stands. For example, for a *Pinus strobus* chronosequence (stands 2, 15, 30, and 65 years old), Peichl and Arain [19] found that fine root (≤2 mm) biomass increased with stand age from 0.2 Mg ha^-1^ in the youngest stand to a peak of 6.2 Mg ha^-1^ in the 30-year-old stand, after which it decreased to 3.5 Mg ha^-1^ in the oldest stand. In a *Pinus sylvestris* chronosequence (stands 15, 35 and 100 years old) Helmisaari et al. [12] found a peak in FRB at the age of 35 (pole stage stand that had just reached canopy closure) and suggested that the time of canopy closure determines FRB maximum. Børja et al. [15] studied stand age influence on FRB to a soil depth of 60 cm in *Picea abies* stands 10, 30, 60, and 120 years old, and revealed that FRB was significantly higher in the 30-yr-old stand and lower in the 10-yr-old stand, than in the older stands (60 and 120 years old). A similar trajectory was found in deciduous forests. For example, Claus and George [13] observed FRB development in *Fagus sylvatica* and *Quercus cerris* forest chronosequences. Their study showed that FRB reached a maximum at an approximate age of 25, and then declined to a steady-state, as forests approached maturity. Analogous patterns of FRB development over stand age were observed by Bakker et al. [20]. They examined a *Fagus sylvatica* chronosequence consisting of stands 9, 26, 82 and 146 years old, and found that FRB at depths of 0–120 cm was significantly higher in the younger stands than in the older stands. However, the FRB changes over stand age might be altered by environmental factors, e.g. [9, 21–23].

Studies on fine root biomass changes over stand age are usually based on a relatively small number of stands varying in age and that may influence the results obtained. However, Finér et al. [24] analyzed a large dataset that included published FRB data obtained by the core method for 36 *Fagus sylvatica* stands (30–250 years), 71 *Picea abies* stands (24–200 years) and 43 *Pinus sylvestris* stands (12–131 years). The mean FRB of beech was 389 ± 206 g m^-2^ and this value was noticeably higher than for spruce (297 g m^-2^) and pine stands (277 g m^-2^). The study showed that in the temperate zone beech FRB decreased with stand age whereas pine root biomass increased with stand age. Fine root biomass expressed on the tree level correlated better with stand structural attributes than on the stand level. The FRB per tree increased with basal area per tree in beech and spruce stands in the temperate zone, and in pine stands in the boreal zone. The data shown in the cited paper partly supported the hypothesis that FRB increases by the time of canopy closure, and either does not increase or decreases thereafter. However, the results clearly show that at the tree level, there exists a strong correlation between the FRB and age. A similar trajectory of FRB changes, based on 218 root studies conducted in boreal forests, was also reported by Yuan and Chen [21]. The authors stated that with increasing stand age, FRB increased until about 70–90 years old for forest stands and then leveled off or decreased. They also suggested that the increase of FRB with stand age appeared to be a consequence of fast above- and below-ground biomass accumulation related to stand development and increased nutrient concentration in the upper soil horizons during stand development. Moreover, in the older stands biomass production was lower and the foliage to non-foliage biomass ratio diminished; this might be related to reduced demands for nutrient and water supply from fine roots. As a consequence, this may lead to decreases in FRB with age in old-growth stands.

The spatial variability of roots is a fundamental ecological phenomenon controlling access to soil resources. The majority of fine root biomass in forest stands is generally found in the humus and upper mineral soil layers. During the early decades after planting, trees allocate more resources to FRB in order to maximize water and nutrient uptake that support fast foliage production [8], and during stand development foliage biomass and production increase, reach a maximum and then decrease, e.g. [25,26]. Stand productivity and the relative amount of tree foliage biomass decreases at mature ages, which consequently reduce the demand for nutrient and water supply from fine roots [12,13,27,28]. Thus, biomass allocation to fine roots decreases with stand age, which has been proven by a majority of studies published so far. However, a small number of studies have compared belowground changes in FRB and/or production across a chronosequence of forest stand ages, e.g. [7,9,11,16–18,28]. In general, as mentioned above, it appears that FRB increases to a maximum with canopy closure. Although mature stands are less dense, they have taller and larger trees than immature stands, and this possibly leads to similar competition among trees [28]. Thus, it has been hypothesized that the effects of stand density changes on root biomass over time could be asymptotic [29]–root density stabilizes below a maximum limit, which approximately corresponds to the canopy closure phase.

In general, FRB is highly concentrated in the uppermost soil layer where the availability of nutrients (soil fertility), nitrogen and organic matter, as well as the activity of soil microbial communities are higher, and FRB decreases with depth [30–37]. For example Yuan and Chen [21] stated that 90% of the FRB occurred in the top 30 cm surface layers (humus and upper mineral soil). However, whether fine root distribution patterns, biomass and morphology, are affected by stand age is not evident from the literature. Fine roots adjust rapidly to changes in nutrient and water supply in the soil. For example, Bakker et al. [20] found that the vertical rooting was significantly shallower in the oldest beech stands (a larger fraction of all roots was found in the top 30 cm of the soil) than in the youngest stands. However, their study on root distribution of *Fagus sylvatica* in a chronosequence consisting of stands 9, 26, 82, and 146 years old showed that the variability in fine root distribution depended more on soil depth and edaphic conditions than on stand age. The authors concluded that root systems can vary in their vertical and horizontal distribution in response to local heterogeneity in soil conditions. This finding was also confirmed by Børja et al. [15] who found that stand age and soil layer had significant effects on FRB in *Picea abies* stands.

The overall lack of studies concerning changes in fine root morphology as stands age is surprising. However, a few studies have emphasized the architectural plasticity of fine roots as an essential adaptation to changing growth conditions with stand development, e.g. [15–18, 38]. For example, specific root length (SRL) and specific root area (SRA) are considered as the two most informative fine root morphological traits with regard to environmental changes. SRL is a measure of the ability of fine roots to proliferate in the soil and SRA is a parameter describing the water and nutrient absorbing area of fine roots per unit dry mass [39]. Specific root length (SRL) is considered a more descriptive parameter than biomass from the viewpoint of soil resource exploitation, and this trait characterizes the economic aspects of root system development very well. As summarized by Cornelissen et al. [40], plants with higher SRL are able to form more extended fine roots for a given dry biomass investment and this is attained by constructing roots with smaller diameter or lower tissue density. The changes in fine root biomass and SRL with stand development are relatively well documented [13–15,41]. Higher SRL permits plants to increase soil volume exploration during younger phases of stand development, while low SRL may indicate difficulties in the acquisition of nutrients and water from the soil, and a slow rate of fine root proliferation [42,43]. Data on fine root morphological changes over stand age are missing in the literature. However, it was found in previously published papers that tree species may differ in their primary strategies for maintaining adequate mineral nutrition, i.e. either through a change of fine root biomass or a change in fine root morphology [44]. The role of fine roots may change along with stand development [9,28].

It was proved in previous studies that soil resource acquisition by fine roots is proportional both to their length and surface area, whereas fine root construction and maintenance cost are proportional to their mass [41,43–45]. However, only a few studies have focused on SRA and SRL changes over stand age. For example, Rosenvald at al. [38] have found that SRA and SRL of *Betula pendula* EcM roots significantly decreased with stand age, whereas fine root tissue density and diameter increased. Both SRA and SRL were also reported to be higher for younger stands of *Betula pendula*, *Cryptomeria japonica* and *Pinus sylvestris* in comparison with older stands [14,16,17,46]. Other attributes of fine root morphology (e.g. total fine root length, surface area, volume and number of fine root tips expressed on a stand area basis) were usually not investigated in forest stands, and a majority of the existing data refers to experiments with potted tree saplings; these results may not be directly extrapolated to forest stand conditions. However, these fine root attributes analyzed at the stand level, may help improve understanding of stand age influence on fine roots on a larger scale.

Our objective was to establish how stand age affects fine root biomass and morphology in stand age sequences of three deciduous tree species with various ecological requirements, i.e. *Quercus robur*, *Fagus sylvatica* and *Alnus glutinosa*. Because the forest stands comprised an age continuum, and the soils and topography among stands of each species were similar, we assumed that the potential differences would be related to stand age. We hypothesized that FRB would increase with stand age until canopy closure and then decrease or remain constant. We also assumed that fine root morphology would vary between stands of different ages. We hypothesized that fine root length, surface area, volume and number of root tips expressed on a stand area basis would increase with stand age. To achieve this we analyzed the variability of FRB (on stand area and tree levels) and fine root morphology throughout each chronosequence at two soil depths (0–15 cm and 16–30 cm).

## Material and Methods

### Study sites

The study was conducted in chronosequences of three tree species: *Quercus robur*– 22 stands, *Fagus sylvatica*– 21 stands and *Alnus glutinosa*– 16 stands. The stands were situated in Piaski (51°48’N, 17°07’E), Gryfino (53°20’N, 17°39’E) and Trzcianka (53°03’N, 16°25’E) Forest Districts (Poland), respectively. The field studies did not involve endangered or protected species according to Polish law. Monospecific oak stands were 11–140 years old, beech stands were 9–140 years old and alder stands were 4–76 years old, thus the chronosequences ranged from young stands to stands that have attained commercial and biological maturity (**Table 1**). The stands grow on typical and homogenous soils for each species: oak stands grow on pseudogley soils composed of loamy sands and sandy clays, beech stands grow on acid, brown soils of weakly loamy sand and sandy loam, whereas alder stands grow on peat soils of fen peat bogs and peat-muck soils. Within each stand we established research sites where only an herbaceous layer was present and there were almost no woody species in the understory (shrub layer cover <5% of the stand area studied). In oak stands the *Molinio caeruleae-Quercetum roboris* plant community was identified and the most frequent herb species were *Molinia caerulea*, *Calamagrostis arundinacea*, *Holcus molli*s and *Trientalis europaea*. In beech stands the *Galio odorati-Fagetum* plant community was found and the most frequent plant species were *Ficaria verna*, *Anemone nemorosa*, *A*. *ranunculoides*, *Milium effusum*, *Melica uniflora* and *Poa nemoralis*. The *Fraxino-Alnetum* community was found within alder stands; the most abundant herb species in alder forests were *Galeobdolon luteum*, *Aegopodium podagraria*, *Ficaria verna*, *Stellaria nemorum*, *Circaea lutetiana*, *Stachys sylvatica* and *Impatiens noli-tangere*. No commercial thinnings were conducted in any of the stands studied within 5 years prior to the study.

**Table 1:** Characteristics of beech, oak and alder stands varying in age. Abbreviations: DBH–diameter at breast height, H–tree height, G–total basal area.

Tree species	Standcharacteristics	Stand age (years) and its characteristics																					
**Beech**	**Age (years)**	**9**	**14**	**19**	**25**	**29**	**35**	**45**	**50**	**60**	**63**	**65**	**70**	**85**	**93**	**95**	**101**	**110**	**116**	**121**	**130**	**140**	
	**DBH (cm)**	0.94	4.10	5.99	8.65	9.57	17.71	15.91	24.74	30.14	22.78	33.82	35.34	39.56	46.45	39.03	49.76	39.20	48.63	44.78	45.01	56.37	
	**H (m)**	3.69	7.17	9.00	13.51	12.78	17.83	20.04	29.48	29.70	25.17	32.18	36.80	40.37	39.54	39.68	38.18	33.49	34.77	40.54	37.86	42.41	
	**Density (trees ha**^**-1**^**)**	53611	8118	4550	4160	2389	792	980	553	413	498	377	297	213	198	369	102	377	232	267	255	134	
	**G (m**^**2**^ **ha**^**-1**^**)**	5.12	14.44	13.77	26.60	18.72	20.42	23.31	28.14	32.05	22.65	35.32	30.02	26.58	34.17	45.18	20.52	42.49	44.43	45.26	41.18	38.56	
**Oak**	**Age (years)**	**11**	**14**	**18**	**23**	**38**	**45**	**49**	**49**	**50**	**62**	**68**	**80**	**85**	**85**	**96**	**100**	**111**	**116**	**120**	**126**	**136**	**140**
	**DBH (cm)**	2.93	2.96	2.08	8.44	15.14	17.43	17.89	19.34	15.25	17.90	23.95	27.64	32.31	31.54	35.85	31.86	30.98	34.85	43.77	42.28	42.70	43.42
	**H (m)**	3.91	4.16	3.77	8.98	15.58	17.83	18.25	19.79	15.90	19.88	23.45	24.50	25.73	27.77	28.27	26.41	21.72	25.47	28.66	30.16	31.75	27.03
	**Density (trees ha**^**-1**^**)**	5920	7647	2768	2212	990	510	713	708	1088	614	316	260	274	320	236	212	286	182	157	170	199	183
	**G (m**^**2**^ **ha**^**-1**^**)**	4.96	6.49	1.22	14.49	18.79	12.75	18.77	22.44	21.12	19.07	15.10	16.11	23.32	26.02	24.26	17.48	22.47	18.12	24.48	24.71	29.37	27.69
**Alder**	**Age (years)**	**4**	**4**	**11**	**12**	**23**	**31**	**36**	**40**	**42**	**46**	**46**	**54**	**61**	**66**	**71**	**76**						
	**DBH (cm)**	1.81	0.84	5.92	7.47	14.74	17.07	18.41	18.88	22.48	22.62	21.38	23.52	32.01	17.91	29.12	28.80						
	**H (m)**	2.64	1.78	8.10	8.87	15.60	15.05	14.21	22.01	22.09	22.73	17.57	24.10	27.20	17.01	28.49	22.12						
	**Density (trees ha**^**-1**^**)**	4800	4700	3733	3138	1416	985	1028	865	349	848	551	593	425	853	625	378						
	**G (m**^**2**^ **ha**^**-1**^**)**	1.83	0.33	11.00	16.01	26.12	24.58	29.26	26.37	14.37	36.06	23.25	27.40	37.84	24.50	43.62	25.78						

Long-term meteorological observations from the closest meteorological stations to the studied stands showed that the mean annual temperature was 8.6°C in oak stands, 8.9°C in beech stands, and 7.2°C in alder stands, whereas mean annual precipitation was 597, 550 and 587 mm in oak, beech and alder stands, respectively. The mean growing season length (calculated as the number of days with mean temperature ≥5°C) was 210–220 days in oak stands, 221–224 days in beech stands and 220 days in alder stands.

### Root data collection

In each stand we installed one plot with area from 0.03 to 1.5 ha; plot area depended on tree density and was adjusted to include at least 200 live trees per plot. Prior to root collection, we measured diameter at breast height (1.3 m) and height of trees in all study plots. Detailed data on stands biometrical characteristics are given in the **Table 1**.

Root biomass was estimated by soil core sampling. Ten randomly selected cores per stand were collected in late July/early August 2009 in oak stands, in August 2010 in beech stands and in late August/early September 2010 in alder stands. Although root samples were collected at different parts of the growing seasons (late July–early September), we harvested root biomass near the peak of foliage development in each chronosequence. Thus, even though the main aim of the study was to analyze fine root biomass and morphology changes over the chronosequence within each tree species separately, we have compared the chronosequences among species. Surprisingly, in our previous study with *Quercus robur* we found that patterns of root morphology were generally similar across all seasons studied, i.e. spring, summer and fall [47].

A cylindrical tube 4.7 cm in diameter and 15 cm long with a sharp edge to cut roots was used as the soil corer (Arts MFg. & Supply, American Falls, Idaho, USA). Roots were collected from two soil depths: 0–15 cm and 16–30 cm. Soil samples with roots were placed in plastic bags, labeled, and stored in a refrigerator (4°C) during sample collection in the field. After the samples were transported to the laboratory they were stored in a cooling chamber (-3°C; a temperature that did not damage the live tissue) until sample preparation.

In the laboratory, the fine roots were manually sorted from coarser roots by sieving over 2 mm sieves and then transferred to floating basins with water [16,17]. We used ≤2 mm diameter threshold to define fine roots so that the data would be comparable to other previously published studies, even though it has been suggested in published papers that the term ‘fine roots’ is a combination of static and dynamic root fractions [1,33,48]. Live roots were separated from dead roots based on distinct morphological criteria for each species, such as the degree of cohesion between the cortex and periderm and root color of the central cylinder [39,49]. We omitted the dead fine root mass from further analyses due to considerable contamination by sand/clay particles. The roots of other plant species than tree species studied (i.e. herbaceous species) were not included in the analyses. Roots of herbaceous plant species were distinguished from tree roots by their smaller diameter, non-lignified structure and lighter color. In case of any doubts, we have compared fine roots of tree species studied with root samples of the most common herbaceous plants also collected during the field studies to avoid any uncertainties.

### Morphological analysis

After separation, the fine roots were placed in a transparent water-filled tray and scanned. The images were analyzed with the program WinRhizo 2003b Basic (Regent Instruments Inc, Quebec, QC, Canada) and an Epson Perfection 3200 PHOTO transmitting light scanner. The fine roots were placed on a scanner in a transparent tray (15 cm × 10 cm) filled with deionised water to allow roots to spread out. The roots were scanned in grey scale at 400 dpi resolution with a filter of 1 mm^2^; the program calculated architectural traits for all the fine roots from a given soil core. The tests by Bauhus and Messier [50] and our prior studies [16,17] using the same technique showed negligible errors in fine root morphological measurements due to root overlap. The digitized root images allowed determination of the following fine root morphological parameters:

- mean root diameter (mm),
- total root length (m m^-2^ of soil),
- total root surface area (m^2^ m^-2^ of soil),
- total root volume (cm^3^ m^-2^ of soil),
- number of root tips (no. m^-2^ of soil),
- fine root tip density (tips m^-1^ root length).

Because the number of root tips measured by WinRhizo may be overestimated as the program counts cut roots as tips also, we counted tips and cut roots for three samples of fine roots for each stand under the microscope to calibrate the results obtained by WhinRhizo [17].

After morphological analysis, the dry biomass of fine roots was determined by drying them at 65°C for at least 48 hours in a forced-air dryer (ULE 600) until constant weight. Root samples were weighed with 0.0001 g accuracy (BP 210 S Sartorius). Dry root biomass as well as root morphological features were used to calculate the following fine root parameters:

- specific fine root tip density (tips g^-1^ roots),
- specific fine root surface area (cm^2^ g^-1^ roots),
- specific fine root length (m g^-1^ roots),
- fine root tissue density (g cm^-3^ fine roots).

Fine root biomass, length, surface area, volume and tip number were also calculated at the tree level by dividing the parameters at the stand level (**S1 Table**) by the stand density shown in **Table 1**.

### Statistical analysis

We used analysis of variance (ANOVA, P>F) to determine differences in means of the biomass and morphological fine root traits among tree stand chronosequences of the three species studied. If significant differences were found, multiple comparisons were carried out based on Tukey’s test (HSD) for equal sample sizes at *P*<0.05; in all cases a null hypothesis was rejected at the 5% level of significance (*P*>0.05). The relationship between tree stand age and fine root biomass as well as root morphological traits was assessed using Generalized Additive Models (GAM). We tested GAM, Generalized Linear Models (GLM) and Linear Regression (LR) and based on Akaike’s Information Criteria (AIC) we chose the best fit model to describe the studied relationships. We also inspected residuals to ensure that best fit model was not an effect of artifacts or outliers. In most cases GAM models fit the data best and had the lowest AIC. Moreover, although LR is better for modeling in cases where explanation of the studied phenomena is more important than predictive power, a simplified linear relationship cannot be easily extended outside the range of data on which model was built. For GAM we used a procedure implemented in the *mgcv* package (https://cran.r-project.org/web/packages/mgcv/), with a thin plate regression spline, which better shows non-linear trends connected with tree stand age [51]. In some cases optimal spline parameters calculated by the program were close to the linear regression, reporting linear or almost linear types of relationships. All independent variables in GAM and ANOVA were log-transformed prior to analyses, due to non-normal distributions which were closest to the log-normal distribution, with the exception of percentage variables, which were transformed according to the C. I. Bliss equation. Statistical tests and analyses were performed using JMP 10.0.0. software (SAS Institute Inc., Cary, NC, USA; http://www.sas.com) and R software [52]. We prepared the database and calculated fine root parameters and mean values of biomass in JMP. ANOVA, GAM regression and data visualization were conducted in R. All the data shown in tables and figures are mean values ± standard error (SE).

## Results

### Fine root biomass

**Fig 1:**
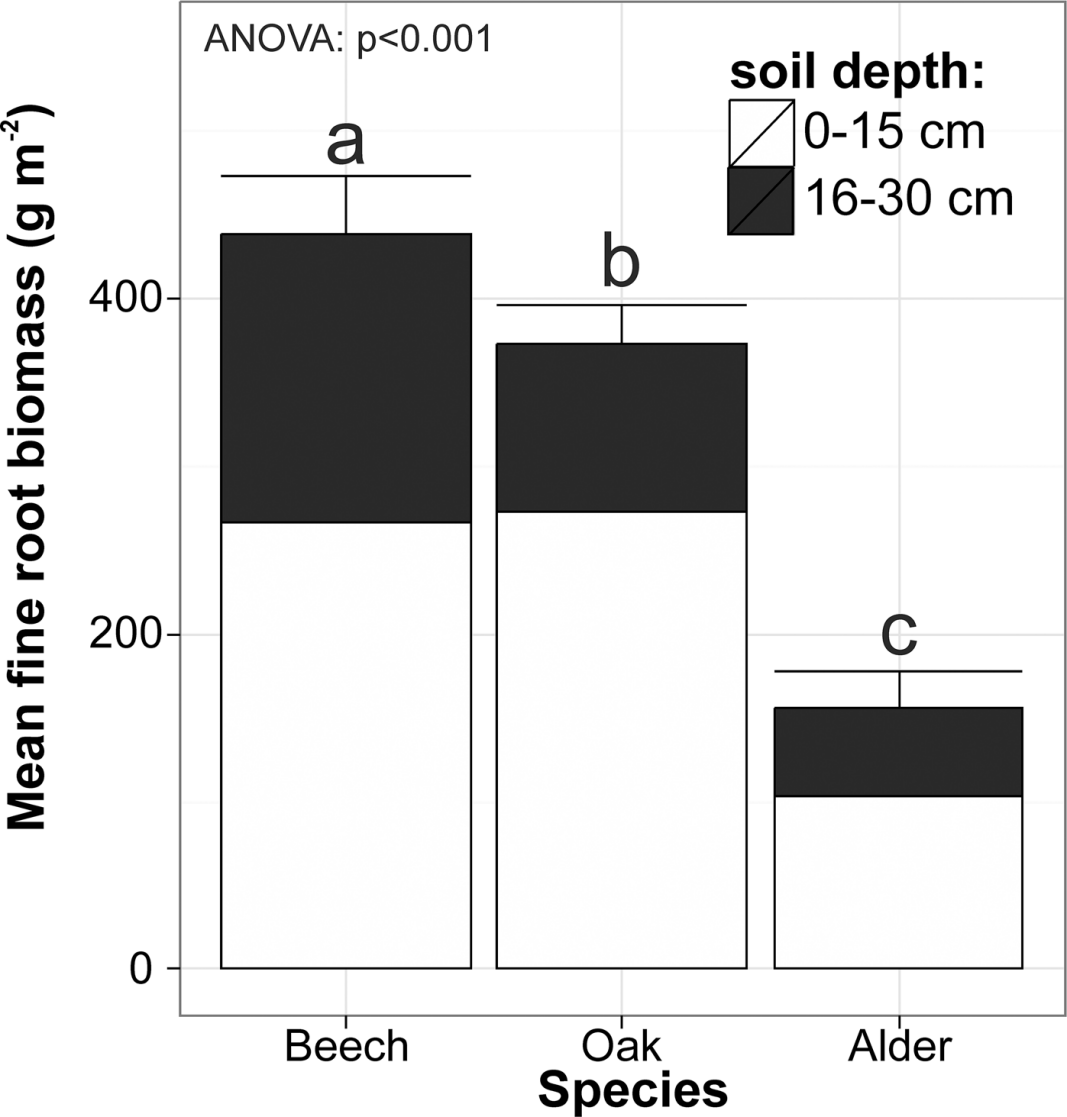
Mean fine root biomass (±SE) from 0–30 cm soil depth for beech, oak and alder stand chronosequences. Analysis of variance was performed to show significance of differences among stand chronosequences for total fine root biomass. Same letters in Tukey’s test show lack of differences among mean values of fine root biomass of beech, oak and alder stands.

There were statistically significant differences among species chronosequences in fine root biomass from 0–30 cm soil depth, expressed on a stand area basis (*P*<0.001; **Fig 1**). Mean FRB was 432 g m^-2^, 371 g m^-2^ and 153 g m^-2^ in beech, oak and alder chronosequences, respectively. On average fine roots in the 0–15 cm soil depth layer constituted as much as 62%, 73% and 69% of the total FRB (0–30 cm) in beech, oak and alder stands, respectively (**S1 Table**).

#### Fagus sylvatica

**Fig 2:**
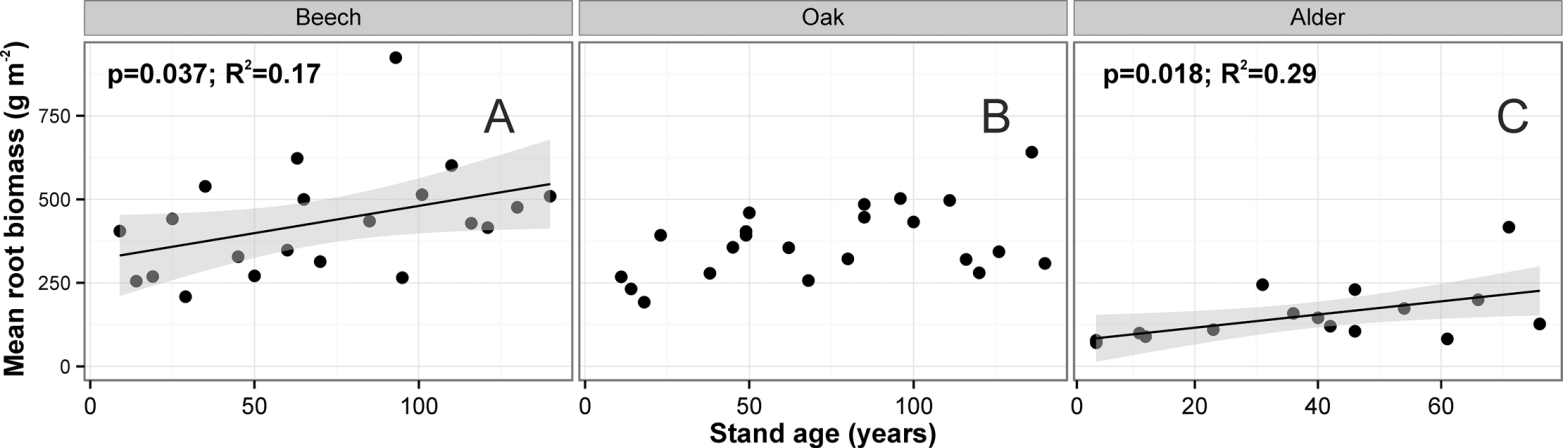
Relationships between stand age and fine root biomass expressed on a stand area basis (g m^-2^) for 0–30 cm soil depth for beech, oak and alder chronosequences. Relationships were modeled using Generalized Additive Models. Shaded area represents the confidence interval of GAM. Points represent mean values for each tree stand, raw datapoints are available in S1 File.

**Fig 3:**
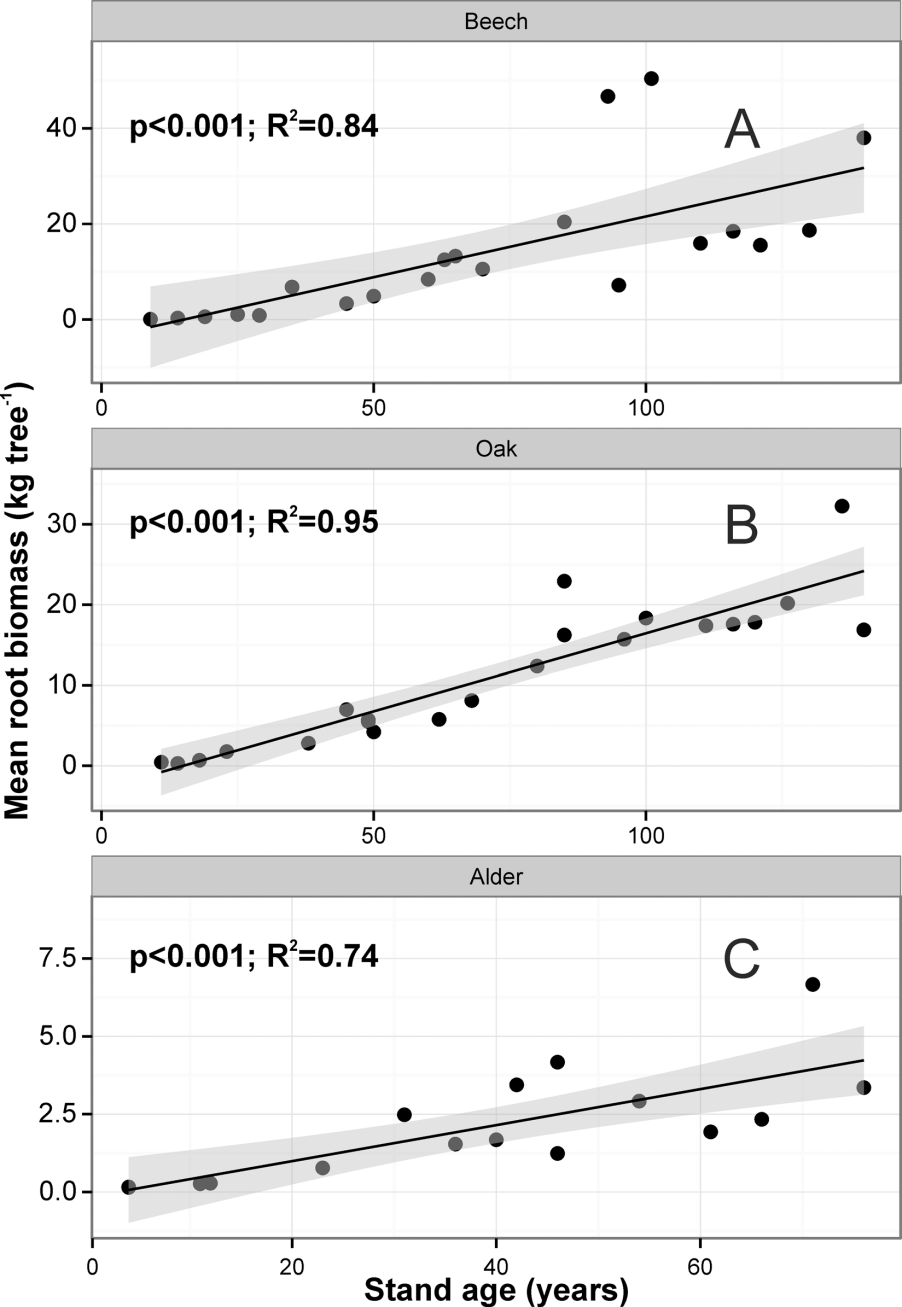
Relationships between stand age and fine root biomass for the average individual tree in each stand (kg tree^-1^) for 0–30 cm soil depth for beech, oak and alder chronosequences, modeled by Generalized Additive Models (GAM). The mean individual tree root biomass was calculated by dividing the respective fine root biomass for each stand per ha (shown in S1 Table) by the number of trees in the stand per ha (shown in Table 1). Shaded area represents the confidence interval of GAM. Points represent mean values for each tree stand, raw datapoints are available in S1 File.

The total FRB in the upper 30 cm of soil ranged from 405 g m^-2^ in the 9-yr-old stand to 509 g m^-2^ in the 140-yr-old (**S1 Table**). The lowest total FRB was found in the 29-yr-old stand (209 g m^-2^), whereas the highest was in the 93-yr-old stand (924 g m^-2^). Fine root biomass in the 0–30 cm soil depth (**Fig 2A**) increased significantly with stand age (r^2^ = 0.17, *P* = 0.037). There was no significant relationship between stand basal area and total FRB expressed on a stand area basis, however total FRB increased linearly with mean tree DBH (r^2^ = 0.19, *P* = 0.027). When fine root biomass from the 0–30 cm soil depth were calculated at the tree level, we found significant relationships with stand age–when stand age increased, mean fine root biomass increased (r^2^ = 0.84, *P<0*.*001*; **Fig 3A**).

We found significant positive relationships between FRB in the upper (0–15 cm) soil layer and stand age (r^2^ = 0.24, *P* = 0.015) and mean tree DBH (r^2^ = 0.27, *P* = 0.009), but there was no significant relationship with stand basal area (*P*>0.05). For the lower soil layer there were no significant relationships between stand age, mean tree DBH and stand basal area and FRB expressed on a stand area basis.

#### Quercus robur

Total FRB in the upper 30 cm of soil ranged from 268 g m^-2^ in the 11-yr-old stand to 308 g m^-2^ in the 140-yr-old (**S1 Table**). The lowest total FRB was found in the 18-yr-old stand (192 g m^-2^) and the 14-yr-old stand (232 g m^-2^), and the highest in the 136-yr-old stand (641 g m^-2^) and the 96-yr-old stand (503 g m^-2^). Total fine root biomass expressed on a stand area basis increased with stand age (r^2^ = 0.23, *P* = 0.053; **Fig 2B**), although the relationship was not statistically significant. Total FRB was positively correlated with mean tree DBH (r^2^ = 0.27, *P* = 0.039) and stand basal area (r^2^ = 0.45, *P*<0.001). When fine root biomass from 0–30 cm soil depth was calculated at the tree level, we found significant relationships with stand age–when stand age increased, mean fine root biomass increased (r^2^ = 0.95, *P*<0.001; **Fig 3B**).

There were no significant relationships between FRB in the upper (0–15 cm) and lower (16–30 cm) soil layers and stand age (*P*>0.05) and mean tree DBH (*P*>0.05), however we noticed that when stand age and mean tree DBH increased, FRB increased at both depths. There were significant positive relationships between stand basal area and FRB in the upper layer (r^2^ = 0.34, *P* = 0.003), however in the lower layer the relationship was not statistically significant (*P>*0.05).

#### Alnus glutinosa

Total FRB biomass in the upper 30 cm of soil ranged from 71 g m^-2^ and 78 g m^-2^ in the 4-yr-old stands to 127 g m^-2^ in the 76-yr-old (**S1 Table**). The lowest total FRB was found in the 4-yr-old stand (71g m^-2^), whereas the highest was in the 71-yr-old stand (417 g m^-2^ Mg ha^-1^). Total FRB per stand area basis increased with stand age (r^2^ = 0.29, *P* = 0.018; **Fig 2C**) and stand basal area (r^2^ = 0.63, *P* = 0.013). When fine root biomass from the 0–30 cm soil depth was calculated at the tree level, we found significant relationships with stand age–when stand age increased, mean fine root biomass increased (r^2^ = 0.74, *P*<0.001; **Fig 3C**).

Fine root biomass in the upper soil layer was not significantly related with stand age (*P>*0.05), but in the lower soil layer the relationship was statistically significant (r^2^ = 0.41, *P* = 0.004). There were no significant relationships between FRB in the upper (0–15 cm) soil depth and mean tree DBH (*P>*0.05), however we found a significant relationship in the lower layer (r^2^ = 0.21, *P* = 0.043). We also found significant relationships between FRB and stand basal area both in upper (r^2^ = 0.36, *P* = 0.008) and lower (r^2^ = 0.65, *P* = 0.009) layers. However, we noticed that even in cases of non-statistically significant relationships, when stand age, mean tree DBH and stand basal area increased, FRB increased at both depths.

### Fine root morphology

The tree species studied differed distinctly in fine root morphology. We found statistically significant differences among species chronosequences in fine root diameter as well as length, surface area, volume, number of root tips (all these values expressed on a stand area basis) and fine root tip density for the upper (**S1 Fig**) and the lower (**S2 Fig**) soil layers. Specific fine root tip density differed significantly among species in the 0–15 cm soil layer (*P* = 0.002; **S1 Fig**), while specific root area differed significantly only in the deeper soil layer (*P* = 0.008; **S2 Fig**). There were no statistically significant differences among the species studied in mean specific root length and root tissue density for the whole chronosequences (**S1 and S2 Figs**).

The lowest fine root length, surface area and volume in the 0–30 cm soil depth, expressed on a stand area basis, occurred in alder stands, with medium values for oak stands and the highest for beech. For example, fine root length in the 0–30 cm soil depth amounted to 5178 m m^-2^, 4724 m m^-2^ and 1642 m m^-2^ in beech, oak and alder stands, respectively. Mean values for beech and oak stands were not significantly different, but both these values differed significantly from fine root length of alder stands (*P*<0.001). In beech stands 61% of total fine root length was found in the upper soil layer, 76% in oak stands and 67% in alder stands (**S1 and S2 Figs**). Total number of root tips on a stand area basis in the 0–30 cm soil depth was not significantly different among beech and oak stands (1131730 vs. 1226002, respectively), however these values were significantly higher (ca. threefold) than mean number of root tips in alder stands (370978 tips m^-2^ soil; *P*<0.001). In the upper soil layer we found 60%, 77% and 66% of total number of root tips in the entire 0–30 cm soil depth for beech, oak and alder stands, respectively (**S1 and S2 Figs**).

#### Fagus sylvatica

**Fig 4:**
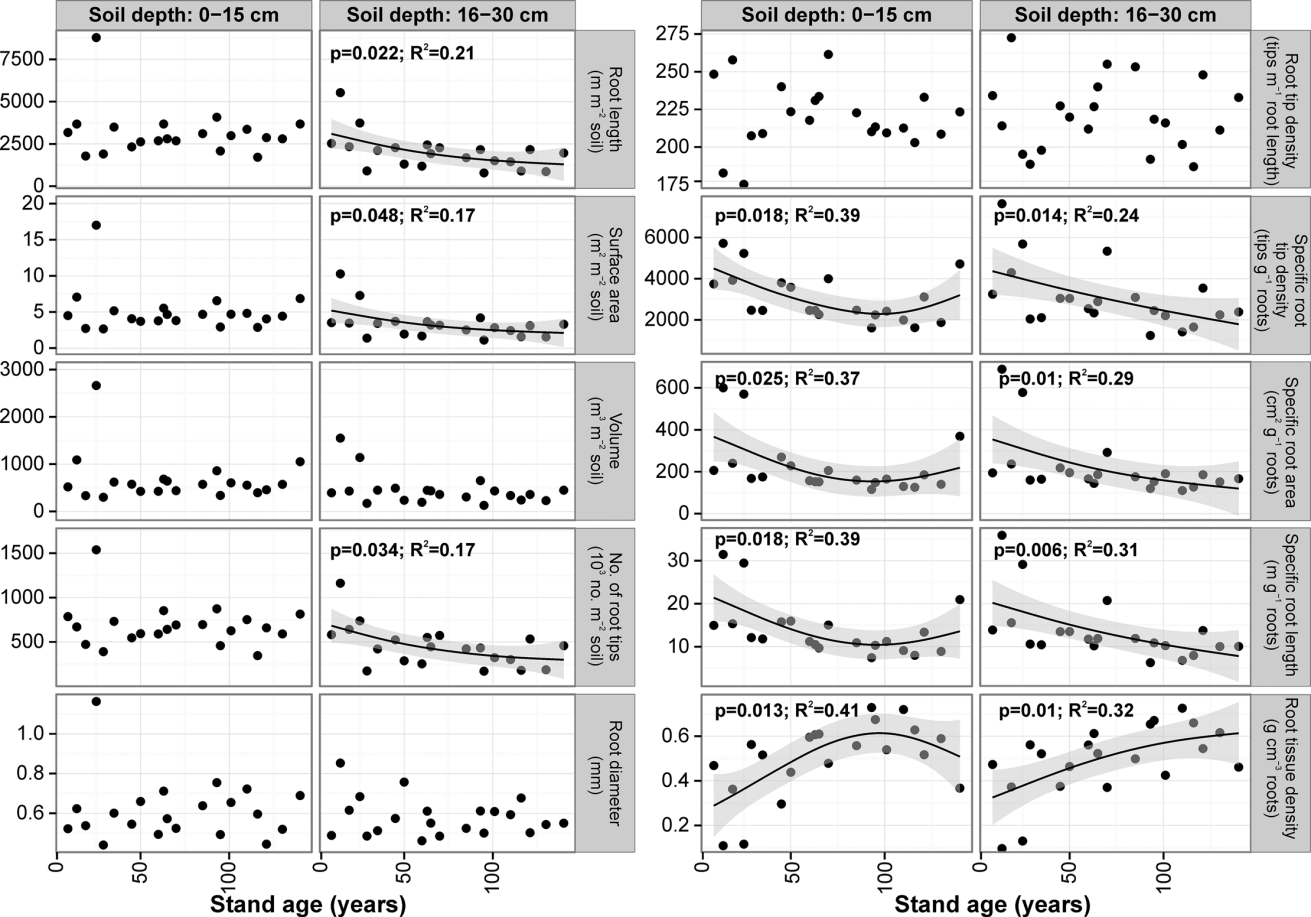
Relationships between stand age and fine root morphological traits for 0–15 cm and 16–30 cm soil depths for the beech stand chronosequence. Relationships were modeled using a Generalized Additive Model. Shaded area represents the confidence interval of GAM. Points represent mean values for each tree stand, raw datapoints are available in S1 File.

Fine root diameter was not significantly related with stand age and did not differ significantly between soil depths–the mean was 0.61 mm (range: 0.44–1.16 mm) for the upper layer and 0.58 mm (range: 0.46–0.85 mm) for the lower layer. For most of the stands we found statistically significant differences in fine root length between the soil layers studied (**S2 Table**). Total fine root length (for both soil depths) was highest in the 25-yr-old stand (12520 m m^-2^ soil) and lowest in the 116-yr-old stand (2614 m m^-2^ soil). In the lower soil layer we found negative relationships between stand age and fine root length (r^2^ = 0.21, *P* = 0.022) and fine root surface area (r^2^ = 0.17, *P* = 0.048) expressed on a stand area basis (**Fig 4**). However, when stand age increased, fine root length proportion in the upper soil layer increased (r^2^ = 0.26, *P* = 0.011). Mean fine root surface area in the upper soil layer ranged from 2.66 to 17.01 m^2^ m^-2^ and in the lower soil layer from 1.12 to 10.27 m^2^ m^-2^, and differed significantly between soil depths for half of the stands (**S2 Table**). In the upper soil layer it averaged 61% of total fine root surface area. Fine root surface area proportion in the upper soil layer increased with stand age (r^2^ = 0.26, *P* = 0.011). Number of root tips on a stand area basis was significantly different among stands in both soil layers (*P*<0.001; **S2 Table**). Mean number of tips in the upper soil layer was ca. 1.5-fold that in the lower soil layer (681670 tips m^-2^ soil vs. 445360 tips m^-2^) and amounted ca. 60% of total number of root tips. When stand age increased, number of fine root tips per 1 m^2^ of 16–30 cm soil depth decreased (r^2^ = 0.17, *P* = 0.034; **Fig 4**). Moreover, we found a statistically significant positive relationship between stand age and proportion of fine root tips in the upper soil layer (r^2^ = 0.22, *P* = 0.017).

For most of the stands studied there were no significant differences in fine root tip density (mean for upper layer: 220 tips m^-1^, mean for lower layer: 221 tips m^-1^), specific root tip density (3054 vs. 3067 tips g^-1^), specific root area (222 vs. 219 cm^2^ g^-1^) and length (14.0 vs. 13.6 m g^-1^) and fine root tissue density (0.50 vs. 0.49 g cm^-3^) between soil depths (**S2 Table**). For the upper soil layer, when stand age increased, specific root tip density (r^2^ = 0.39, *P* = 0.018), specific root area (r^2^ = 0.37, *P* = 0.025), and specific root length decreased (r^2^ = 0.39, *P* = 0.018), whereas fine root tissue density increased (r^2^ = 0.41, *P* = 0.013) up to an age of 100 years. For the lower soil layer, when stand age increased, specific root tip density (r^2^ = 0.24, *P* = 0.014), specific root area (r^2^ = 0.29, *P* = 0.010) and specific root length decreased (r^2^ = 0.31, *P* = 0.006), whereas fine root tissue density increased (r^2^ = 0.32, *P* = 0.010). For 0–30 cm of soil depth specific root length decreased with increasing stand age (r^2^ = 0.40, *P* = 0.005). The shape of the regression line in this soil layer was straighter than in the upper soil layer (**Fig 4**).

**Fig 5:**
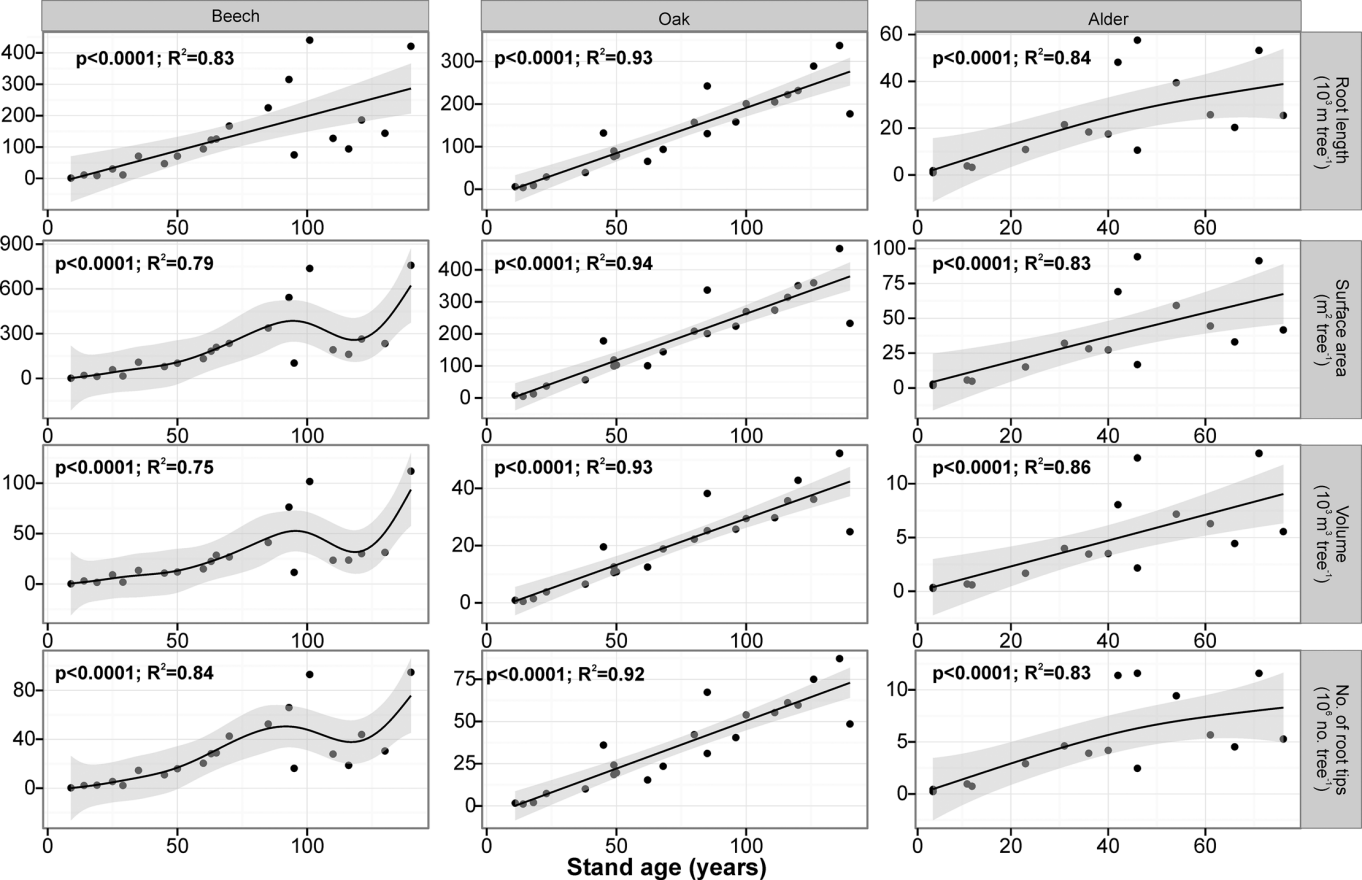
Relationships between stand age and fine root length, surface area, volume and number of root tips for the average individual tree in each stand for 0–30 cm soil depth for beech, oak and alder chronosequences modeled by Generalized Additive Models (GAM). The mean individual fine root indices were calculated by dividing the particular root traits values for each stand per ha (shown in S2, S3 and S4 Tables) by the number of trees in the stand per ha (shown in Table 1). Shaded area represents the confidence interval of GAM. Points represent mean values for each tree stand, raw datapoints are available in S1 File.

When age of beech stands increased, mean fine root length, surface area, volume and number of root tips in 0–30 cm of soil expressed on tree stand area decreased, but the relationships were not statistically significant (*P*>0.05). However, the above mentioned fine root parameters calculated on a per tree basis, had a statistically significant increase with increasing stand age (**Fig 5**).

#### Quercus robur

**Fig 6:**
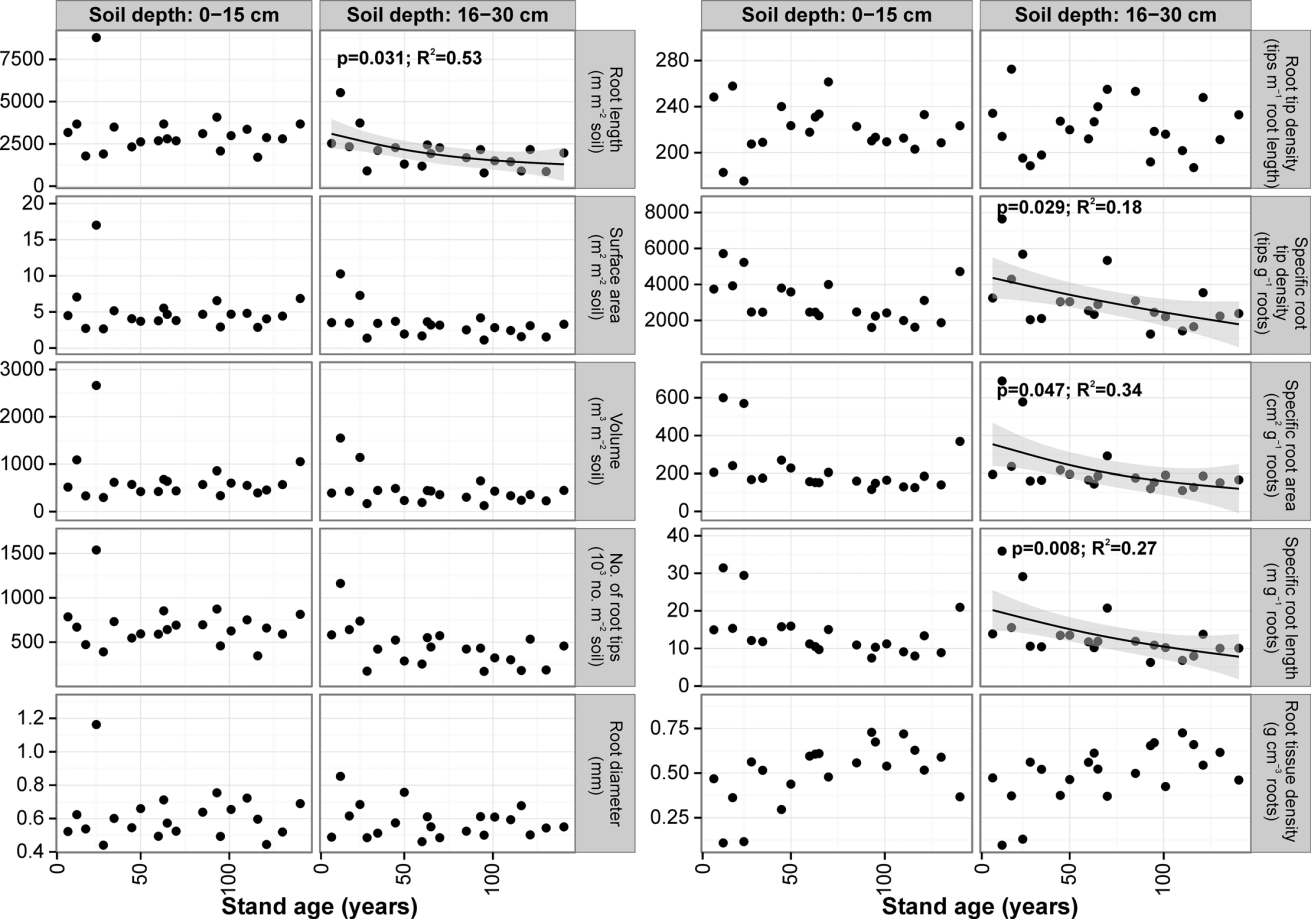
Relationships between stand age and fine root morphological traits for 0–15 cm and 16–30 cm soil depths for the oak stand chronosequence. Relationships were modeled using Generalized Additive Models. Shaded area represents the confidence interval of GAM. Points represent mean values for each tree stand, raw datapoints are available in S1 File.

Mean diameter was 0.45 mm (range: 0.40–0.58 mm) for the 0–15 cm depth, and 0.46 mm (range: 0.41–0.55 mm) for the 16–30 cm depth. For most of the stands we found statistically significant differences in fine root length between the soil layers studied (**S3 Table**). Total fine root length (for both soil depths) was the highest in the 50-yr-old stand (8468 m m^-2^ soil) and the lowest in the 18-yr-old stand (2451 m m^-2^ soil). We found no relationship between stand age and fine root length expressed on a stand area basis in the upper soil layer (*P*>0.05), whereas in the lower soil layer this relationship was statistically significant (r^2^ = 0.53, *P* = 0.031; **Fig 6**). This relationship was generally deceasing, with two peaks in the 46 year old and 100 year old stands. Mean fine root surface area ranged from 2.30 to 8.67 m^2^ m^-2^ in the upper soil layer and from 0.98 to 2.50 m^2^ m^-2^ in the lower layer, and differed significantly between soil depths for most of the stands (**S3 Table**). Fine root surface area in the upper soil layer averaged 74% of total fine root surface area. There was no significant relationship between stand age and fine root surface area calculated for either soil layer (*P*>0.05). This relationship was generally deceasing, with two peaks in the 46 year old and 100 year old stands. We found statistically significant differences between soil depths in number of fine root tips for all stands older than 18-yrs (**S3 Table**). Mean number of tips in the upper soil layer for the whole chronosequence was ca. threefold higher than in the lower soil layer (943400 tips m^-2^ soil vs. 284700 tips m^-2^) and amounted ca. 75% of total number of root tips.

For most of the stands studied we found no significant differences in fine root traits between soil layers: root tip density (mean for upper layer: 258 tips m^-1^, mean for lower layer: 242 tips m^-1^), specific root tip density (3634 vs. 3356 tips g^-1^), specific root area (189 vs. 180 cm^2^ g^-1^), specific root length (13.9 vs. 13.4 m g^-1^) and fine root tissue density (0.54 vs. 0.60 g cm^-3^) (**S3 Table**). We found no significant relationships between stand age and the above mentioned fine root morphological traits calculated for 0–15 cm (**Fig 6**). For the lower soil layer (16–30 cm), there were negative, statistically significant relationships between stand age and specific root tip density (r^2^ = 0.18, *P* = 0.029), specific root area (r^2^ = 0.34, *P* = 0.047) and specific root length (r^2^ = 0.27, *P* = 0.008; **Fig 6**). For 0–30 cm soil depth specific root length decreased with increasing stand age (r^2^ = 0.49, *P* = 0.013).

There were no statistically significant relationships between oak stand age, mean fine root length, surface area, volume and number of root tips expressed on a tree stand area (*P*>0.05). However, above mentioned fine root parameters calculated on a per tree basis increased linearly and statistically significantly with increasing stand age (**Fig 5**).

#### Alnus glutinosa

**Fig 7:**
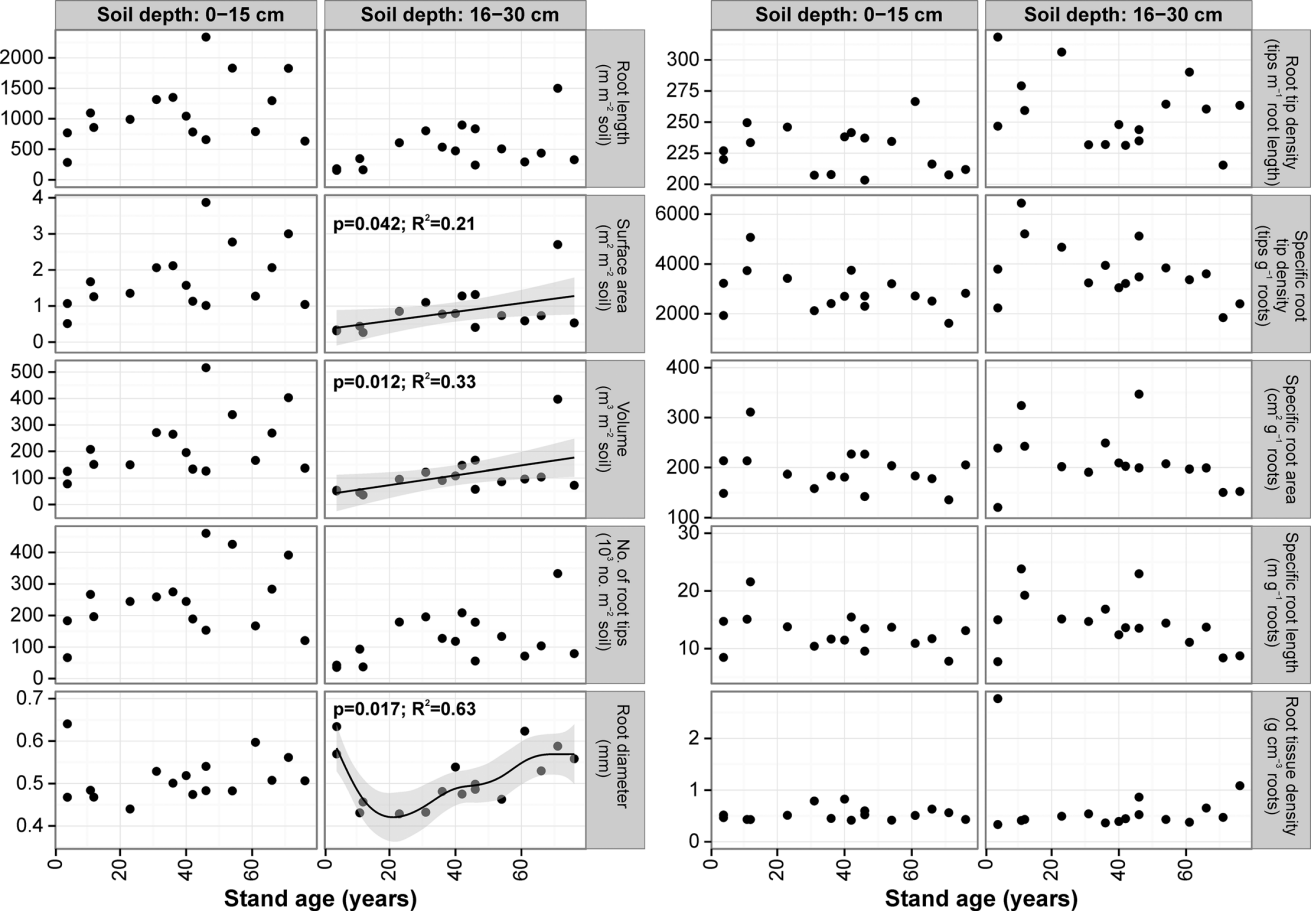
Relationships between stand age and fine root morphological traits for 0–15 cm and 16–30 cm soil depths for the alder stand chronosequence. Relationships were modeled using Generalized Additive Models. Shaded area represents the confidence interval of GAM. Points represent mean values for each tree stand, raw datapoints are available in S1 File.

Mean fine root diameter for 0–15 cm and 16–30 cm soil depths were the same and equaled 0.51 mm (range for 0–15 cm: 0.44–0.64 mm, and for 16–30 cm: 0.43–0.63 mm). Total fine root length (0–30 cm) was highest in the 71-yr-old stand (3327 m m^-2^ soil) and lowest in the 4-yr-old stand (466 m m^-2^ soil). In the upper soil layer mean fine root length ranged from 285 to 2340 m m^-2^ and in the lower soil layer from 154 to 1500 m m^-2^. Although the mean fine root length increased with stand age, the relationships were not statistically significant for any soil depths (*P*>0.05; **Fig 7**). Mean fine root surface area in the upper soil layer ranged from 0.51 to 3.87 m^2^ m^-2^ soil and in the lower soil layer from 0.27 to 2.70 m^2^ m^-2^ soil and differed significantly between soil depths for half of the stands (**S4 Table**). Fine root surface area in the upper soil layer averaged 70% of total fine root surface area. There was no significant relationship between stand age and fine root surface area for 0–15 cm soil depth (P>0.05), however this relationship was positive and statistically significant for the 16–30 cm soil depth (r^2^ = 0.21, *P* = 0.042; **Fig 7**). A similar relationship was also noted for fine root volume in the 16–30 cm soil layer (r^2^ = 0.33, *P* = 0.012). Mean number of root tips on a stand area basis was significantly different among stands for both soil layers for half of the stands studied (**S4 Table**). Mean number of tips in 0–30 cm soil depth for all the stands, was 362300 tips m^-2^ and 69% of total number of tips were found in the upper soil layer. There were no significant relationships between stand age and any of the fine root parameters for the 0–15 cm soil layer (**Fig 7**).

For most of the stands studied we found no significant differences in fine root tip density between soil layers (mean for upper layer: 228 tips m^-1^, mean for lower layer: 258 tips m^-1^), specific root tip density (2891 vs. 3718 tips g^-1^), specific root area (194 vs. 215 cm^2^ g^-1^), specific root length (12.7 vs. 14.4 m g^-1^) and fine root tissue density (0.53 vs. 0.66 g cm^-3^) (**S4 Table**). We found no significant relationship between stand age and the above mentioned fine root morphological traits calculated for the 0–15 cm soil depth, however in the 16–30 cm soil depth there were statistically significant negative relationships between stand age and fine root volume (r^2^ = 0.33, *P* = 0.012), and fine root surface area (r^2^ = 0.21, *P* = 0.042). For the 0–30 cm soil layer the relationship between specific fine root length and stand age was negative, but not statistically significant (*P*>0.05).

There were not statistically significant relationships between alder stand age, mean fine root length, surface area, volume and number of root tips expressed on a tree stand area basis, however these relationships are positive (*P*>0.05). The above mentioned fine root parameters calculated on a per tree basis increased statistically significantly with increasing stand age (**Fig 5**).

## Discussion

The results of our study show that FRB increases with stand age and this pattern was confirmed for all three tree species studied. In contrast to previously published studies suggesting that maximum FRB is reached at the canopy closure stage of stand development, we found almost linear increases of FRB per stand area basis, over stand ages of the chronosequences. Fine root biomass in the 0–30 cm soil depth (expressed on a stand area basis) increased significantly for oak and beech chronosequences and also showed clear increasing trend for alder stands. We did not observe a FRB peak in the canopy closure stage. This may indicate that the age of monocultures is an important factor that determines FRB dynamics, however it may be related to other stand characteristics such as stand density, stand structure, basal area, phase of stand development, aboveground biomass, and previous management practices, among others [22,53–55].

The average fine root biomasses in our study were within the ranges reported for other temperate forests. For example, global estimates of fine root (≤2 mm) biomass by Jackson et al. [56] were 440 g m^-2^ for temperate deciduous and 500 g m^-2^ for coniferous forests. FRB in temperate forests reviewed by Vogt et al. [2] ranged from 75 to 1633 g m^-2^ (526 g m^-2^ on average) while Noguchi et al. [57] found that FRB of deciduous broad-leaved Japanese forests ranged from 240–1160 g m^-2^. Our data for the particular tree species studied also confirmed the previous findings regarding high FRB variability, which may be related to tree species ecology and thus species-specific adaptations to the local environment [58]. For example, FRB in the top 40 cm of soil ranged from 320 to 470 g m^-2^ in 110–152 year-old beech forests in northwestern Germany [59], while in our study mean FRB in the top 30 cm of soil averaged 486 g m^-2^ and ranged from 415 to 601 g m^-2^ for 110–140 year-old stands. Hertel et al. [60] found that FRB ranged from 289 to 704 g m^-2^ in 87-142-year-old beech stands growing in northwestern Germany (on average: 482 g m^-2^) while in our study mean FRB for the same soil depth and similar stand age (93–140 years old) averaged 432 g m^-2^ and ranged from 265 to 924 g m^-2^. Another study [36] found that FRB (upper 20 cm of soil) averaged 272 g m^-2^ in 146-year-old beech stands (range: 248–304 g m^-2^) while in our study FRB averaged 309 g m^-2^ in the upper 15 cm of soil in the oldest stand of the chronosequence studied (140 years old). Our FRB data are also within the pan-European range for beech stands summarized by Finér et al. [24] where, for 30-250-year-old stands (mean: 111 years), it ranged from 116 to 960 g m^-2^ and averaged 389 g m^-2^. Leuschner and Hertel [61] reported mean FRB of 470 g m^-2^ for beech forests (range: 118–960 g m^-2^) aged 30–151 years. However, only a few studies have been published reporting the fine root data of oak and even fewer for alder so far. For example, Curiel Yuste et al. [62] found that biomass of fine roots (≤2 mm) was 2.0 Mg ha^-1^ and biomass of small roots (2–5 mm) was 1.8 Mg ha^-1^ for 30 cm soil depth in 67-year-old pedunculate oak in Belgium; our data have shown higher FRB in the upper 30 cm of soil in stands with similar age. Leuschner and Hertel [61] reported that FRB of sessile oak stands from 20–131 years old averaged 316 g m^-2^ and ranged from 163 to 415 g m^-2^. There is a lack of literature on fine root biomass for alder stands. Persson and Stadenberg [63] examined a 34 year-old stand of *Alnus glutinosa* (1600 trees/ha) in Sweden and found that FRB (<1 mm) amounted to 21 g m^-2^ in the humus horizon (0–25 cm) and 29 g m^-2^ in mineral soil down to 40 cm, whereas roots with <10 mm diameter had masses of 113 and 267 g m^-2^, respectively. Aosaar et al. [64] estimated FRB in young grey alder (*Alnus incana*) stands growing on abandoned agricultural land at 55, 57 and 81 g m^-2^ to a depth of 40 cm, thus similar to our data for black alder.

Mean FRB in 0–30 cm soil depth of the beech chronosequence (432 g m^-2^) was significantly higher than for oak (371 g m^-2^) and alder (153 g m^-2^) stands. The differing age range of beech and oak stands in comparison with alder stands may have been a source of differences in mean FRB, however it is unlikely to find alder stands older than 80 years since older trees suffer from heart rot [65]. Thus, it might be assumed that for each species we analyzed the full possible chronosequence from young stands to adult and mature stands. However, significantly lower FRB in alder stands may be also a result of soil properties–alder stands were growing on peat soils, which were more humid and with distinctly higher organic matter content in the upper 30 cm of soil than soils in the beech and oak stands. Black alder is a N-fixing and water demanding tree species with high juvenile growth rates, and the root systems of alder are well adapted to wet soils [65]. Also, the nitrogen demand of alders is higher than that of the other tree species studied; moreover, leaf litter of this species is extremely rich in nitrogen due to low nitrogen retranslocation from senescing leaves, and it mineralizes very fast, leading to high nitrogen storage in the upper soil layers [66–68]. Under high nutrient status and soil moisture, trees may decrease their carbon investment in explorative fine root length growth, and this is also clearly shown by the lower FRB per stand area basis [69]. For example, in our study the total fine root length (0–30 soil depth) amounted to 5178 m m^-2^, 4724 m m^-2^ and 1642 m m^-2^ in beech, oak and alder chronosequences, respectively. At the same time we found no statistically significant differences among species chronosequences in mean specific fine root length, at either of the soil depths studied. When we expressed fine root length per tree, we noticed almost the same relationship between stand age and mean fine root length for beech and oak stands, but distinctly lower values for alder stands. The increase of FRB on a stand area basis may also be a result of necromass increase in the upper soil organic layers (organic horizon) in ageing stands [13,16,17]. As a stand ages, greater amounts of decomposed litter enrich the soil in nutrients and this creates more suitable conditions for further root growth and development in the upper soil horizons [70]. This may partly explain the increasing amounts of FRB as stands age and develop.

The effect of stand age on fine root biomass is still controversial. Nevertheless, it is generally suggested that FRB increases until the canopy closure stage is reached, after which it levels off [21], decreases [13,21], or increases [24]. In most of the studies describing FRB changes over stand age it was suggested that the belowground growing space was already filled and closed by the age of canopy closure [8,9,12,17,19,46]. However, Yuan and Chen [21], based on the published literature, noticed that FRB changed with stand development and continued to increase in stands up to 70 and 90 years old for deciduous and coniferous stands, respectively. The cited authors stated that FRB increased until the canopy transition stage of stand development and then leveled off or decreased at the gap dynamics or old growth stage. However, we found no such a peak in any of the species studied. For example, Claus and George [13] studied a *Fagus sylvatica* chronosequence and found that the maximum FRB is reached in ca. 25-year-old stands, and after this peak FRB biomass started to decline. In mature stands FRB reached a steady-state. The cited authors described a clear decline in FRB (0–30 cm soil depth, plus the organic layer) with age for beech stands as follows: 5.3–6.4 Mg ha^-1^ for the 15- and 30-year-old stands and 2.4–3.3 Mg ha^-1^ for 62- and 111-year-old stands, respectively. Our data for the same tree species and soil depth showed the opposite trend, e.g. 2.55–2.08 Mg ha^-1^ for the 14- and 29-year-old stands and 6.23–6.04 Mg ha^-1^ for 63- and 110-year-old stands, respectively. Leuschner and Hertel [61] found a negative (but not statistically significant) relationship between stand age (x) and FRB (y) for broadleaf stands. For example, for *Fagus* stands this relationship was as follows: y = 857–2.54x. In another study, Finér et al. [24] have shown that FRB in beech stands expressed on a stand area basis decreased with stand age (thus opposite to our results), whereas when FRB was expressed at the tree level, it increased with stand age and basal area per tree. This relationship was also shown in our study. We compared estimates of beech FRB (y) versus stand age (x) on stand area basis and tree basis, using equations published by Finér et al. [24] and similar (linear regression) equations developed based on data collected for our study. Mean FRB in 30-, 50-, 70- and 100-year-old stands estimated by Finér et al. [24] equations were 552, 489, 457 and 407 g m^-2^, whereas the values estimated by our equations were 366, 399, 431 and 480 g m^-2^, respectively. This comparison clearly suggests that tree density might be an important factor influencing fine root biomass at the stand level; even though individual older trees have higher FRB than younger trees, the FRB for the whole stand might decrease over age because tree density decreases, e.g. [15,54,71]. When FRB was expressed on a tree basis, it amounted to 6.1, 8.4, 10.8 and 14.3 kg tree^-1^ using the Finér et al. [24] equations, and 3.8, 8.9, 13.9 and 21.6 kg tree^-1^ using equations developed in this study (again for 30-, 50-, 70- and 100-year-old beech stands). Finér et al. [24] based their analyses on published data where stand age ranged from 30 to 250 years (9–140 years in our study), mean DBH ranged from 7 to 54 cm (0.9–56.4 cm), mean tree height ranged from 11 to 34 m (3.7–42.4 m), mean stand density ranged from 108 to 4768 trees ha^-1^ (102–63511 trees ha^-1^) and stand basal area ranged from 17 to 52 m^2^ ha^-1^ (5.1–45.3 m^2^ ha^-1^ in our chronosequence). These clear differences in stand characteristics may have important influence on standing FRB; moreover in the cited paper no young stands (up to 30 years old) were included in the analyses since no published data on fine root biomass were found.

In this study we did not find intensive increases of FRB (on a stand area basis) in any of the chronosequences during the youngest stages of stand development. In our previous study conducted in Scots pine stands growing on a reclaimed lignite mine spoil heap (age range: 6–20 years old) we found very dynamic increases in FRB across the chronosequence; in the oldest stand it was ca. 4 times higher than in the youngest one [16]. These changes were parallel to foliage biomass and leaf area index changes in the same stands [26]. In another study conducted on post-agricultural stands (6-47-years-old) we found that the highest increase of FRB occurred between the 6- and 10-year-old stands, and after that a steady level of FRB was reached in stands 10–47 years old. It was concluded that Scots pine FRB reaches a constant value much earlier than suggested in previously conducted studies, but at the same stage of stand development, e.g. at the time of canopy closure [17].

Our data have also shown that fine root biomass expressed at the tree level was better correlated with stand structural attributes than root biomass expressed at the stand level; this relationship was proved for all three species studied (**Fig 4**). A previous study [24] also showed different relationships at tree and stand levels; the relationship of stand age on FRB was positive at the tree level, but negative at the stand level. The suggestion of Helmisaari et al. [72], that fine root biomass and morphology should be estimated at an average tree level, was partially supported in our study for all the species studied. We found some relationships between stand age and basal area and fine root characteristics at the stand level, but the relationships were highly significant when the calculations were based on the average tree in the stand. The proportion of the variation explained by the model was relatively high in each species. This suggests that the mean tree approach may be more representative in even-aged pure stands than when expressed on a stand area level [42,72,73]. However, Chang et al. [74] have shown for black locust plantations (8 and 30 years old) that FRB in the top 20 cm layer and entire soil profile (0–100 cm) increased both expressed on a stand area basis and tree level basis.

To completely recognize fine root response to changes in stand age and thus stand growing conditions, root biomass examined alone is not sufficiently indicative of the functional potential of the root system. Alterations in root systems may also occur without any change in total root biomass [75]. Our study has shown not only significant differences in FRB by age within each tree species, but also significant differences in fine root morphology over the particular chronosequence. Moreover, our data have shown that fine root length, surface area, volume and number of fine root tips per stand area basis (m^2^) differed significantly among tree species studied. All of these parameters were significantly higher in beech and oak chronosequences in comparison with alder stands, at both soil depths studied. Although the fine root indices expressed on a stand area basis differed among stands, they were generally not related with stand age. The upper soil horizon in our study was densely exploited by fine roots in all the species studied (e.g. fine root biomass, fine root length). Fine roots in forest stands usually have huge length per area, which allows efficient exploration of the soil and rapid colonization of resource-rich microsites [76,77].

Our study has reported a negative relationship between stand age and SRL for beech, oak and alder (which was statistically insignificant) chronosequences, however this trend was not linear, due to lower values of SRL at the first years of stand life and higher variability of SRL in first and last points of chronosequences (**Figs 4, 6 and 7**). This higher variability may be connected with different dynamics of juvenile tree growth and different history of tree stand development, e.g. connected with former thinning or mortality events in case of older tree stands, which differed in density and basal area (**Table 1**). Roots with high SRL are considered to be less expensive to produce by the plant and increasing SRL allows plants to increase the soil volume exploited per unit biomass invested in fine roots [39,41,78]. This is especially important in young, newly developed stands. For example Ostonen et al. [79] found significant differences in fine root morphology among black alder stands aged 27 and 4 years–mean short-root morphological characteristics for the 27 year old stand were as follows: specific root area– 103.8 m^2^ kg^-1^, specific root length– 85.1 m g^-1^ and root tissue density– 106.7 kg m^-3^, whereas in the 4 year old stand these values were: specific root area– 155.2 m^2^ kg^-1^, specific root length– 126.4 m g^-1^ and root tissue density– 69.6 kg m^-3^. In our previous study with Scots pine growing on a lignite mine spoil heap we also found a decreasing SRL with increasing stand age from 22.4 m g^-1^ in a 6-year-old stand to 12.3 m g^-1^ in a 20-year-old stand [16]. In another study with Scots pine growing on abandoned agricultural sites we found that fine roots from younger stands (6- and 10-years old) had significantly greater SRL than in the older stands (16-, 28- and 47-years old) [17]. Higher SRL values at younger tree age were also revealed in other studies, e.g. for *Quercus alba* and *Acer saccharum*, where SRL of roots of first and second orders was higher for 1-year-old seedlings than for older trees [80]. The mean SRL was also smaller in mature stands than in younger stands of *Betula pendula* and *Pinus sylvestris* [46]. In our study mean SRL was not significantly different among the tree species chronosequences. For example, in the upper soil layer (0–15 cm) it was 13.9 m g^-1^ for beech and oak chronosequences and 12.7 m g^-1^ for alder stands. We also found no significant differences in SRL by soil depth for any of the species studied. In contrast to our results, Kalliokoski et al. [46] have found that SRL of *Betula pendula* fine roots were generally higher in the humus layer than in the mineral soil in sapling, pole and mature developmental stages. In another study, Børja et al. [15] found that stand age had no effect on SRL of the finest root fraction (<1 mm), however for 1–2 mm and 2–5 mm roots, the effect of stand age was clear, with the highest mean values in the pole stage. Also opposite to our results, the cited authors noticed that soil layer had a significant effect on SRL for all root fractions, with the highest values in mineral soil (0–20 cm) and the lowest at soil depth of 40–60 cm. Moreover, they found no effect of stand age on root tip density for any tree root fraction, with means of about 2 tips cm^-1^ root length. However, the cited results suggested that root tips are more abundant in deeper soil layers independently of stand age. In our study we found significant differences among tree species chronosequences in fine root tip density; for example, in the 0–15 cm soil layer, there were 258 tips m^-1^ for oak stands, which differed significantly from beech and alder stands (220 and 228 tips m^-1^, respectively). Within a particular chronosequence fine root tip density was in general not significantly influenced by soil depth.

The results shown in this paper indicate high fine root plasticity. In conclusion, our study showed that stand age affects both fine root biomass and morphology. We found that fine root biomass increased continuously with increasing stand age of beech, oak and alder chronosequences. Thus, our findings did not support the hypothesis that the highest fine root biomass would be related to the canopy closure stage of stand development. We think that other site and stand variables might be important in fine root biomass production and changes over stand age, for example stand density or standing biomass (and its allocation). This was clearly shown when fine root biomass data were expressed on a tree area basis (thus including decreasing stand density with increasing stand age). The two main strategies of fine root adaptation to differences in site and stand features over stand age suggested by Ostonen et al. [41,44,79] might be identified: trees enhance their carbon investment to increase fine root biomass and at the same time they change fine root morphology to increase their nutrient and water uptake from the soil and thus improve their efficiency. We hypothesized that fine root length, surface area, volume and number of root tips expressed on a stand area basis would increase with stand age. This relationship was found only for alder stands–when stand age increases, fine root length, surface area, volume and number of root tips per stand area basis also increases. For beech stands all these relationships were negative whereas for oak stands we found a linear increase of the fine root parameters studied in the youngest stands, but they reached a steady state in stands ca. 50 or more years old. This result clearly shows different strategies of fine root development over stand age in the species studied. Thus, estimating changes in belowground biomass and production is essential for our understanding of fundamental patterns and processes during forest ecosystem development. Although only limited datasets are currently available, this data have provided valuable insight into fine root biomass and morphology of beech, oak and alder stands. Fine root biomass and morphology varies greatly with tree species and age.

## Supporting Information

S1 Fig Mean (±SE) fine root morphological traits for beech, oak and alder chronosequences for 0–15 cm soil depth. One-way ANOVAs were performed separately for each fine root trait studied to show significance of differences among tree species. Same letters indicate a lack of statistically significant differences between analyzed stand chronosequences according to Tukey’s posteriori test (*P*<0.05).
(TIF)

S2 Fig Mean (±SE) fine root morphological traits for beech, oak and alder chronosequences for 16–30 cm soil depth. One-way ANOVAs were performed separately for each fine root trait studied to show significance of differences among tree species. Same letters indicate a lack of statistically significant differences between analyzed stand chronosequences according to Tukey’s posteriori test (*P*<0.05).
(TIF)

S1 File Raw datapoints of measured parameters. Spreadsheet in.csv format with all raw datapoints of measured parameters and meta-data about tree stands, where roots were sampled.
(CSV)

S1 Table Mean (±SE) fine root biomass in beech, oak and alder stands differing in age for 0–15 cm, 16–30 cm and 0–30 cm soil depths. (DOCX)

S2 Table Morphological traits of fine roots harvested in beech stands differing in age for 0–15 cm and 16–30 cm soil depths. One-way ANOVAs were performed separately for the root traits studied to show significance of differences in fine root morphology between soil depths in each stand. Abbreviation: n.s. means not significantly different.
(DOCX)

S3 Table Morphological traits of fine roots harvested in oak stands differing in age for 0–15 cm and 16–30 cm soil depths. One-way ANOVAs were performed separately for the root traits studied to show significance of differences in fine root morphology between soil depths in each stand. Abbreviation: n.s. means not significantly different.
(DOCX)

S4 Table Morphological traits of fine roots harvested in alder stands differing in age for 0–15 cm and 16–30 cm soil depths. One-way ANOVAs were performed separately for the root traits studied to show significance of differences in fine root morphology between soil depths in each stand. Abbreviation: n.s. means not significantly different.
(DOCX)
