# Supplementary material for: Tree Age Effects on Fine Root Biomass and Morphology over Chronosequences of Fagus sylvatica, Quercus robur and Alnus glutinosa Stands
Source: PLoS One. 2016 Feb 9;11(2):e0148668. doi: 10.1371/journal.pone.0148668 (PMC4747558; doi:10.1371/journal.pone.0148668)
Supplement: S1 Table — (DOCX) [file pone.0148668.s004.docx]

**S1 Table**

| **Soil depth**  **(cm)** | **Trait** | **Root biomass (Mg ha^-1^)** | | | | | | | | | | | | | | | | | | | | | |
| --- | --- | --- | --- | --- | --- | --- | --- | --- | --- | --- | --- | --- | --- | --- | --- | --- | --- | --- | --- | --- | --- | --- | --- |
|  |  | **Beech stands** | | | | | | | | | | | | | | | | | | | | | |
| **Stand age** | | **9** | **14** | **19** | **25** | **29** | **35** | **45** | **50** | **60** | **63** | **65** | **70** | **85** | **93** | **95** | **101** | **110** | **116** | **121** | **130** | **140** |  |
| **0-15** | **Mean** | 2.23 | 1.21 | 1.21 | 2.97 | 1.71 | 3.24 | 1.55 | 1.66 | 2.44 | 3.83 | 3.11 | 1.95 | 2.90 | 5.63 | 2.03 | 3.43 | 3.80 | 2.41 | 2.30 | 3.36 | 3.09 |  |
|  | **SE** | 0.32 | 0.15 | 0.16 | 0.36 | 0.17 | 0.44 | 0.31 | 0.12 | 0.27 | 0.42 | 0.50 | 0.34 | 0.26 | 0.62 | 0.31 | 0.79 | 0.42 | 0.37 | 0.31 | 0.33 | 0.56 |  |
| **16-30** | **Mean** | 1.82 | 1.47 | 1.48 | 1.44 | 0.90 | 2.15 | 1.73 | 1.05 | 1.04 | 2.66 | 1.89 | 1.19 | 1.44 | 3.61 | 0.79 | 1.71 | 2.21 | 1.87 | 2.05 | 1.41 | 2.00 |  |
|  | **SE** | 0.18 | 0.23 | 0.21 | 0.37 | 0.11 | 0.26 | 0.22 | 0.15 | 0.14 | 0.40 | 0.35 | 0.19 | 0.18 | 0.33 | 0.15 | 0.38 | 0.33 | 0.43 | 0.45 | 0.50 | 0.28 |  |
| **0-30** | **Mean** | 4.05 | 2.55 | 2.69 | 4.41 | 2.08 | 5.39 | 3.28 | 2.71 | 3.48 | 6.23 | 5.00 | 3.13 | 4.35 | 9.24 | 2.65 | 5.14 | 6.01 | 4.28 | 4.15 | 4.76 | 5.09 |  |
|  | **SE** | 0.35 | 0.18 | 0.33 | 0.22 | 0.31 | 0.43 | 0.42 | 0.22 | 0.25 | 0.57 | 0.58 | 0.37 | 0.36 | 0.68 | 0.31 | 0.71 | 0.49 | 0.72 | 0.61 | 0.48 | 0.71 |  |
|  | | | | | | | | | | | | | | | | | | | | | | | |
|  | | **Oak stands** | | | | | | | | | | | | | | | | | | | | | |
| **Stand age** | | **11** | **14** | **18** | **23** | **38** | **45** | **49** | **49** | **50** | **62** | **68** | **80** | **85** | **85** | **96** | **100** | **111** | **116** | **120** | **126** | **136** | **140** |
| **0-15** | **Mean** | 1.43 | 1.57 | 1.56 | 3.36 | 1.87 | 2.43 | 3.46 | 2.76 | 3.43 | 2.81 | 1.95 | 2.46 | 3.23 | 3.46 | 3.58 | 2.91 | 3.81 | 2.27 | 2.05 | 2.37 | 5.42 | 1.71 |
|  | **SE** | 0.26 | 0.26 | 0.32 | 0.37 | 0.23 | 0.50 | 0.49 | 0.35 | 0.46 | 0.45 | 0.40 | 0.17 | 0.27 | 0.39 | 0.36 | 0.41 | 0.37 | 0.33 | 0.28 | 0.18 | 0.47 | 0.31 |
| **16-30** | **Mean** | 1.25 | 0.75 | 0.36 | 0.56 | 1.02 | 1.26 | 0.47 | 1.27 | 1.30 | 0.74 | 0.61 | 0.76 | 1.24 | 1.39 | 1.45 | 1.41 | 1.16 | 0.93 | 0.75 | 1.06 | 0.99 | 1.37 |
|  | **SE** | 0.19 | 0.16 | 0.15 | 0.23 | 0.19 | 0.22 | 0.11 | 0.25 | 0.24 | 0.14 | 0.14 | 0.16 | 0.12 | 0.31 | 0.20 | 0.30 | 0.18 | 0.28 | 0.16 | 0.15 | 0.11 | 0.19 |
| **0-30** | **Mean** | 2.68 | 2.32 | 1.92 | 3.92 | 2.79 | 3.56 | 3.92 | 4.04 | 4.60 | 3.55 | 2.57 | 3.22 | 4.46 | 4.85 | 5.02 | 4.32 | 4.97 | 3.20 | 2.80 | 3.43 | 6.41 | 3.08 |
|  | **SE** | 0.29 | 0.35 | 0.28 | 0.47 | 0.34 | 0.54 | 0.45 | 0.55 | 0.51 | 0.42 | 0.47 | 0.23 | 0.24 | 0.52 | 0.37 | 0.67 | 0.47 | 0.37 | 0.32 | 0.22 | 0.53 | 0.36 |
|  | | | | | | | | | | | | | | | | | | | | | | | |
|  |  | **Alder stands** | | | | | | | | | | | | | | | | | | | | | |
| **Stand age** | | **4** | **4** | **11** | **12** | **23** | **31** | **36** | **40** | **42** | **46** | **46** | **54** | **61** | **66** | **71** | **76** |  |  |  |  |  |  |
| **0-15** | **Mean** | 0.72 | 0.38 | 0.80 | 0.67 | 0.77 | 1.77 | 1.19 | 1.05 | 0.55 | 0.71 | 1.85 | 1.39 | 0.60 | 1.31 | 2.19 | 0.62 |  |  |  |  |  |  |
|  | **SE** | 0.27 | 0.07 | 0.24 | 0.14 | 0.15 | 0.60 | 0.23 | 0.27 | 0.11 | 0.10 | 0.27 | 0.15 | 0.17 | 0.38 | 0.40 | 0.18 |  |  |  |  |  |  |
| **16-30** | **Mean** | 0.21 | 0.33 | 0.20 | 0.22 | 0.40 | 0.68 | 0.39 | 0.41 | 0.72 | 0.34 | 0.45 | 0.34 | 0.28 | 0.82 | 1.98 | 0.65 |  |  |  |  |  |  |
|  | **SE** | 0.08 | 0.09 | 0.07 | 0.15 | 0.10 | 0.10 | 0.14 | 0.08 | 0.17 | 0.11 | 0.12 | 0.06 | 0.10 | 0.43 | 0.58 | 0.29 |  |  |  |  |  |  |
| **0-30** | **Mean** | 0.78 | 0.70 | 0.99 | 0.89 | 1.10 | 2.44 | 1.58 | 1.45 | 1.20 | 1.05 | 2.30 | 1.73 | 0.82 | 1.99 | 4.17 | 1.27 |  |  |  |  |  |  |
|  | **SE** | 0.24 | 0.12 | 0.30 | 0.17 | 0.19 | 0.61 | 0.27 | 0.28 | 0.20 | 0.16 | 0.30 | 0.13 | 0.24 | 0.62 | 0.80 | 0.40 |  |  |  |  |  |  |
