# Supplementary material for: Tree Age Effects on Fine Root Biomass and Morphology over Chronosequences of Fagus sylvatica, Quercus robur and Alnus glutinosa Stands
Source: PLoS One. 2016 Feb 9;11(2):e0148668. doi: 10.1371/journal.pone.0148668 (PMC4747558; doi:10.1371/journal.pone.0148668)
Supplement: S2 Table — One-way ANOVAs were performed separately for the root traits studied to show significance of differences in fine root morphology between soil depths in each stand. Abbreviation: n.s. means not significantly different. (DOCX) [file pone.0148668.s005.docx]

**S2 Table**

| **Fine root raits** | **Soil depth**  **(cm)** | **Stan age (years)** | | | | | | | | | | | | | | | | | | | | |
| --- | --- | --- | --- | --- | --- | --- | --- | --- | --- | --- | --- | --- | --- | --- | --- | --- | --- | --- | --- | --- | --- | --- |
|  |  | **9** | **14** | **19** | **25** | **29** | **35** | **45** | **50** | **60** | **63** | **65** | **70** | **85** | **93** | **95** | **101** | **110** | **116** | **121** | **130** | **140** |
| **Diameter**  **(mm)** | **0-15** | 0.52 | 0.62 | 0.54 | 1.16 | 0.44 | 0.60 | 0.55 | 0.66 | 0.49 | 0.71 | 0.57 | 0.52 | 0.64 | 0.75 | 0.49 | 0.65 | 0.72 | 0.60 | 0.44 | 0.52 | 0.69 |
|  | **16-30** | 0.49 | 0.85 | 0.62 | 0.68 | 0.49 | 0.51 | 0.57 | 0.76 | 0.46 | 0.61 | 0.55 | 0.48 | 0.52 | 0.61 | 0.50 | 0.61 | 0.59 | 0.68 | 0.50 | 0.54 | 0.55 |
|  | **ANOVA** | n.s. | n.s. | n.s. | <0.01 | n.s. | n.s. | n.s. | n.s. | n.s. | n.s. | n.s. | n.s. | n.s. | n.s. | n.s. | n.s. | n.s. | n.s. | n.s. | n.s. | n.s. |
| **Length**  **(m m^-2^ soil)** | **0-15** | 3178 | 3674 | 1785 | 8786 | 1906 | 3496 | 2325 | 2623 | 2686 | 3678 | 2803 | 2682 | 3107 | 4079 | 2079 | 2989 | 3364 | 1713 | 2872 | 2801 | 3678 |
|  | **16-30** | 2531 | 5532 | 2338 | 3734 | 904 | 2114 | 2280 | 1306 | 1186 | 2439 | 1927 | 2272 | 1684 | 2163 | 788 | 1505 | 1448 | 901 | 2166 | 867 | 1962 |
|  | **ANOVA** | n.s. | n.s. | n.s. | <0.01 | <0.01 | <0.05 | n.s. | <0.01 | <0.01 | <0.05 | n.s. | n.s. | <0.01 | <0.01 | <0.01 | <0.05 | <0.01 | <0.05 | n.s. | <0.01 | <0.01 |
| **Surface area**  **(m^2^ m^-2^ soil)** | **0-15** | 4.50 | 7.06 | 2.72 | 17.01 | 2.66 | 5.16 | 4.06 | 3.71 | 3.77 | 5.53 | 4.66 | 3.81 | 4.68 | 6.56 | 2.92 | 4.69 | 4.81 | 2.87 | 4.04 | 4.43 | 6.86 |
|  | **16-30** | 3.53 | 10.27 | 3.47 | 7.28 | 1.38 | 3.43 | 3.70 | 1.95 | 1.69 | 3.64 | 3.20 | 3.17 | 2.52 | 4.18 | 1.12 | 2.83 | 2.43 | 1.58 | 3.10 | 1.54 | 3.30 |
|  | **ANOVA** | n.s. | n.s. | n.s. | <0.01 | <0.01 | <0.05 | n.s. | <0.01 | <0.01 | <0.05 | n.s. | n.s. | <0.01 | <0.05 | <0.01 | n.s. | <0.01 | n.s. | n.s. | <0.01 | <0.01 |
| **Volume**  **(cm^3^ m^-2^ soil)** | **0-15** | 519 | 1089 | 333 | 2662 | 300 | 620 | 575 | 424 | 426 | 683 | 642 | 439 | 573 | 860 | 338 | 604 | 555 | 396 | 458 | 572 | 1051 |
|  | **16-30** | 394 | 1550 | 430 | 1140 | 170 | 449 | 491 | 236 | 193 | 447 | 435 | 360 | 305 | 650 | 128 | 433 | 336 | 241 | 359 | 228 | 449 |
|  | **ANOVA** | n.s. | n.s. | n.s. | <0.01 | <0.05 | n.s. | n.s. | <0.01 | <0.01 | n.s. | n.s. | n.s. | <0.05 | n.s. | <0.01 | n.s. | <0.05 | n.s. | n.s. | <0.01 | <0.05 |
| **No. of root tips**  **(×10^3^ m^-2^ soil)** | **0-15** | 785 | 669 | 472 | 1539 | 390 | 732 | 546 | 593 | 590 | 853 | 642 | 692 | 696 | 873 | 458 | 626 | 751 | 346 | 659 | 590 | 814 |
|  | **16-30** | 582 | 1162 | 641 | 737 | 173 | 421 | 523 | 287 | 253 | 551 | 445 | 573 | 422 | 432 | 170 | 322 | 301 | 180 | 533 | 186 | 457 |
|  | **ANOVA** | n.s. | n.s. | n.s. | <0.01 | <0.01 | <0.05 | n.s. | <0.01 | <0.01 | <0.05 | n.s. | n.s. | <0.05 | <0.01 | <0.01 | <0.05 | <0.01 | <0.05 | n.s. | <0.01 | <0.01 |
| **Root tip density**  **(tips m^-1^ fine roots)** | **0-15** | 248 | 183 | 258 | 175 | 207 | 209 | 240 | 223 | 218 | 231 | 234 | 261 | 223 | 210 | 213 | 209 | 213 | 203 | 233 | 209 | 223 |
|  | **16-30** | 234 | 214 | 273 | 195 | 189 | 198 | 227 | 220 | 212 | 227 | 240 | 255 | 253 | 192 | 218 | 216 | 202 | 187 | 248 | 211 | 233 |
|  | **ANOVA** | n.s. | <0.01 | n.s. | <0.05 | n.s. | n.s. | n.s. | n.s. | n.s. | n.s. | n.s. | n.s. | n.s. | n.s. | n.s. | n.s. | n.s. | n.s. | n.s. | n.s. | n.s. |
| **Specific root tip density**  **(tips g^-1^ fine roots)** | **0-15** | 3742 | 5716 | 3922 | 5227 | 2467 | 2457 | 3800 | 3583 | 2460 | 2459 | 2258 | 3997 | 2470 | 1608 | 2240 | 2417 | 1991 | 1621 | 3108 | 1871 | 4716 |
|  | **16-30** | 3246 | 7640 | 4295 | 5683 | 2041 | 2105 | 3042 | 3042 | 2543 | 2335 | 2885 | 5335 | 3089 | 1240 | 2451 | 2205 | 1415 | 1651 | 3541 | 2243 | 2381 |
|  | **ANOVA** | n.s. | n.s. | n.s. | n.s. | n.s. | n.s. | <0.05 | n.s. | n.s. | n.s. | n.s. | n.s. | n.s. | n.s. | n.s. | n.s. | n.s. | n.s. | n.s. | n.s. | n.s. |
| **Specific root area**  **(cm^2^ g^-1^ fine roots)** | **0-15** | 206 | 600 | 241 | 570 | 168 | 175 | 270 | 228 | 157 | 153 | 152 | 206 | 160 | 116 | 149 | 165 | 130 | 126 | 185 | 140 | 369 |
|  | **16-30** | 195 | 689 | 237 | 578 | 161 | 164 | 219 | 196 | 166 | 145 | 187 | 292 | 176 | 120 | 153 | 191 | 110 | 127 | 186 | 152 | 167 |
|  | **ANOVA** | n.s. | n.s. | n.s. | n.s. | n.s. | n.s. | <0.05 | n.s. | n.s. | n.s. | n.s. | <0.05 | n.s. | n.s. | n.s. | n.s. | n.s. | n.s. | n.s. | n.s. | n.s. |
| **Specific root length**  **(m g^-1^ fine roots)** | **0-15** | 15.0 | 31.5 | 15.3 | 29.4 | 12.1 | 11.8 | 15.8 | 15.9 | 11.2 | 10.5 | 9.7 | 15.0 | 10.9 | 7.4 | 10.3 | 11.2 | 9.1 | 8.0 | 13.4 | 8.9 | 20.9 |
|  | **16-30** | 13.9 | 35.9 | 15.6 | 29.1 | 10.6 | 10.4 | 13.5 | 13.5 | 11.8 | 10.2 | 11.9 | 20.7 | 11.9 | 6.3 | 10.9 | 10.2 | 6.8 | 7.9 | 13.8 | 10.0 | 10.1 |
|  | **ANOVA** | n.s. | n.s. | n.s. | n.s. | n.s. | n.s. | n.s. | n.s. | n.s. | n.s. | n.s. | n.s. | n.s. | n.s. | n.s. | n.s. | n.s. | n.s. | n.s. | n.s. | n.s. |
| **Root tissue density**  **(g cm^-3^ fine roots)** | **0-15** | 0.47 | 0.11 | 0.36 | 0.12 | 0.56 | 0.52 | 0.30 | 0.44 | 0.60 | 0.61 | 0.61 | 0.48 | 0.56 | 0.73 | 0.67 | 0.54 | 0.72 | 0.63 | 0.52 | 0.59 | 0.37 |
|  | **16-30** | 0.47 | 0.10 | 0.37 | 0.13 | 0.56 | 0.52 | 0.38 | 0.46 | 0.56 | 0.61 | 0.52 | 0.37 | 0.50 | 0.65 | 0.67 | 0.42 | 0.73 | 0.66 | 0.54 | 0.62 | 0.46 |
|  | **ANOVA** | n.s. | n.s. | n.s. | n.s. | n.s. | n.s. | n.s. | n.s. | n.s. | n.s. | n.s. | n.s. | n.s. | n.s. | n.s. | n.s. | n.s. | n.s. | n.s. | n.s. | n.s. |
