# Supplementary material for: Tree Age Effects on Fine Root Biomass and Morphology over Chronosequences of Fagus sylvatica, Quercus robur and Alnus glutinosa Stands
Source: PLoS One. 2016 Feb 9;11(2):e0148668. doi: 10.1371/journal.pone.0148668 (PMC4747558; doi:10.1371/journal.pone.0148668)
Supplement: S3 Table — One-way ANOVAs were performed separately for the root traits studied to show significance of differences in fine root morphology between soil depths in each stand. Abbreviation: n.s. means not significantly different. (DOCX) [file pone.0148668.s006.docx]

**S3 Table**

| **Fine root traits** | **Soil depth (cm)** | **Stand age (years)** | | | | | | | | | | | | | | | | | | | | | |
| --- | --- | --- | --- | --- | --- | --- | --- | --- | --- | --- | --- | --- | --- | --- | --- | --- | --- | --- | --- | --- | --- | --- | --- |
|  |  | **11** | **14** | **18** | **23** | **38** | **45** | **49** | **49** | **50** | **62** | **68** | **80** | **85** | **85** | **96** | **100** | **111** | **116** | **120** | **126** | **136** | **140** |
| **Diameter**  **(mm)** | **0-15** | 0.49 | 0.40 | 0.52 | 0.40 | 0.44 | 0.42 | 0.42 | 0.42 | 0.41 | 0.49 | 0.58 | 0.43 | 0.50 | 0.45 | 0.45 | 0.42 | 0.43 | 0.45 | 0.49 | 0.41 | 0.45 | 0.40 |
|  | **16-30** | 0.48 | 0.41 | 0.47 | 0.43 | 0.48 | 0.44 | 0.41 | 0.49 | 0.45 | 0.55 | 0.41 | 0.41 | 0.49 | 0.45 | 0.48 | 0.47 | 0.45 | 0.45 | 0.49 | 0.41 | 0.43 | 0.48 |
|  | **ANOVA** | n.s. | n.s. | n.s. | n.s. | <0.05 | n.s. | n.s. | <0.05 | n.s. | n.s. | <0.01 | n.s. | n.s. | n.s. | n.s. | n.s. | n.s. | n.s. | n.s. | n.s. | n.s. | <0.01 |
| **Length**  **(m m^-2^ soil)** | **0-15** | 1985 | 2029 | 1623 | 5710 | 2365 | 4563 | 5560 | 4065 | 6689 | 3094 | 2189 | 3234 | 2618 | 3972 | 3827 | 3433 | 4761 | 2997 | 2572 | 3655 | 5737 | 2307 |
|  | **16-30** | 1612 | 1054 | 827 | 702 | 1416 | 1920 | 861 | 1367 | 1780 | 928 | 775 | 848 | 974 | 1156 | 1231 | 1299 | 1096 | 1050 | 1070 | 1259 | 971 | 926 |
|  | **ANOVA** | n.s. | n.s. | n.s. | <0.01 | <0.01 | <0.05 | <0.01 | <0.01 | <0.01 | <0.01 | <0.01 | <0.01 | <0.01 | <0.01 | <0.01 | <0.01 | <0.01 | <0.05 | <0.01 | <0.01 | <0.01 | <0.01 |
| **Surface area**  **(m^2^ m^-2^ soil)** | **0-15** | 2.75 | 2.51 | 2.30 | 7.22 | 3.30 | 6.23 | 7.33 | 5.17 | 8.67 | 4.72 | 3.51 | 4.28 | 4.05 | 5.47 | 5.37 | 4.58 | 6.36 | 4.25 | 3.87 | 4.56 | 7.96 | 2.86 |
|  | **16-30** | 2.10 | 1.27 | 1.19 | 0.98 | 2.12 | 2.50 | 1.14 | 1.89 | 2.35 | 1.46 | 1.05 | 1.14 | 1.48 | 1.65 | 1.80 | 1.77 | 1.48 | 1.47 | 1.64 | 1.55 | 1.30 | 1.39 |
|  | **ANOVA** | n.s. | n.s. | n.s. | <0.01 | <0.05 | <0.05 | <0.01 | <0.01 | <0.01 | <0.01 | <0.01 | <0.01 | <0.01 | <0.01 | <0.01 | <0.01 | <0.01 | <0.05 | <0.01 | <0.01 | <0.01 | <0.01 |
| **Volume**  **(cm^3^ m^-2^ soil)** | **0-15** | 309 | 252 | 270 | 736 | 368 | 688 | 776 | 533 | 913 | 582 | 481 | 455 | 508 | 615 | 611 | 494 | 687 | 485 | 469 | 460 | 897 | 285 |
|  | **16-30** | 224 | 124 | 138 | 111 | 256 | 266 | 121 | 216 | 255 | 186 | 115 | 123 | 185 | 194 | 212 | 200 | 162 | 165 | 204 | 155 | 141 | 169 |
|  | **ANOVA** | n.s. | n.s. | n.s. | <0.01 | n.s. | <0.05 | <0.01 | <0.01 | <0.01 | <0.01 | <0.05 | <0.01 | <0.01 | <0.01 | <0.01 | <0.01 | <0.01 | <0.05 | <0.01 | <0.01 | <0.01 | <0.05 |
| **No. of root tips**  **(×10^3^ m^-2^ soil)** | **0-15** | 510 | 549 | 362 | 1465 | 627 | 1250 | 1507 | 1024 | 1649 | 738 | 541 | 890 | 637 | 1092 | 1032 | 938 | 1312 | 817 | 700 | 954 | 1493 | 665 |
|  | **16-30** | 450 | 297 | 194 | 161 | 342 | 518 | 220 | 290 | 442 | 205 | 202 | 208 | 217 | 333 | 264 | 332 | 267 | 297 | 238 | 321 | 242 | 222 |
|  | **ANOVA** | n.s. | n.s. | n.s. | <0.01 | <0.01 | <0.05 | <0.01 | <0.01 | <0.01 | <0.01 | <0.01 | <0.01 | <0.01 | <0.01 | <0.01 | <0.01 | <0.01 | <0.05 | <0.01 | <0.01 | <0.01 | <0.01 |
| **Root tip density**  **(tips m^-1^ fine roots)** | **0-15** | 243 | 273 | 219 | 257 | 261 | 277 | 269 | 238 | 240 | 236 | 255 | 270 | 236 | 262 | 264 | 269 | 273 | 269 | 271 | 248 | 257 | 288 |
|  | **16-30** | 253 | 290 | 205 | 220 | 233 | 259 | 271 | 184 | 228 | 210 | 278 | 257 | 224 | 280 | 209 | 244 | 236 | 273 | 221 | 246 | 248 | 246 |
|  | **ANOVA** | n.s. | n.s. | n.s. | <0.05 | n.s. | n.s. | n.s. | <0.05 | n.s. | <0.05 | n.s. | n.s. | n.s. | n.s. | <0.01 | n.s. | <0.05 | n.s. | <0.01 | n.s. | n.s. | <0.05 |
| **Specific root tip density**  **(tips g^-1^ fine roots)** | **0-15** | 3411 | 3554 | 2886 | 4445 | 3531 | 5499 | 4500 | 3407 | 6872 | 2648 | 3186 | 3594 | 1962 | 3005 | 2879 | 3339 | 3465 | 3390 | 3327 | 4058 | 2865 | 4117 |
|  | **16-30** | 3391 | 4158 | 4201 | 3153 | 3433 | 4342 | 8646 | 1997 | 3407 | 2641 | 4899 | 3659 | 1923 | 3033 | 1902 | 2602 | 2468 | 3022 | 3350 | 3147 | 2617 | 1828 |
|  | **ANOVA** | n.s. | n.s. | n.s. | n.s. | n.s. | n.s. | n.s. | <0.05 | n.s. | n.s. | n.s. | n.s. | n.s. | n.s. | <0.05 | n.s. | n.s. | n.s. | n.s. | n.s. | n.s. | <0.01 |
| **Specific root area**  **(cm^2^ g^-1^ fine roots)** | **0-15** | 200 | 156 | 182 | 215 | 185 | 261 | 218 | 179 | 393 | 170 | 178 | 174 | 125 | 155 | 149 | 164 | 168 | 175 | 187 | 193 | 151 | 174 |
|  | **16-30** | 176 | 166 | 281 | 191 | 217 | 207 | 406 | 146 | 187 | 196 | 202 | 166 | 122 | 140 | 126 | 132 | 134 | 152 | 225 | 147 | 135 | 107 |
|  | **ANOVA** | n.s. | n.s. | <0.05 | n.s. | n.s. | n.s. | n.s. | n.s. | n.s. | n.s. | n.s. | n.s. | n.s. | n.s. | n.s. | <0.01 | n.s. | n.s. | n.s. | n.s. | n.s. | <0.01 |
| **Specific root length**  **(m g^-1^ fine roots)** | **0-15** | 14.1 | 12.8 | 12.9 | 17.1 | 13.5 | 19.8 | 16.5 | 14.0 | 28.8 | 11.2 | 12.0 | 13.1 | 8.1 | 11.2 | 10.7 | 12.3 | 12.6 | 12.5 | 12.3 | 15.6 | 10.9 | 14.4 |
|  | **16-30** | 13.2 | 14.0 | 19.7 | 14.8 | 14.6 | 16.1 | 33.1 | 10.1 | 14.0 | 12.6 | 16.5 | 13.5 | 8.3 | 10.3 | 8.7 | 10.1 | 9.9 | 10.9 | 14.8 | 12.1 | 10.3 | 7.3 |
|  | **ANOVA** | n.s. | n.s. | <0.05 | n.s. | n.s. | n.s. | n.s. | n.s. | n.s. | n.s. | n.s. | n.s. | n.s. | n.s. | n.s. | n.s. | n.s. | n.s. | n.s. | n.s. | n.s. | <0.01 |
| **Root tissue density**  **(g cm^-3^ fine roots)** | **0-15** | 0.46 | 0.66 | 0.60 | 0.49 | 0.51 | 0.39 | 0.47 | 0.56 | 0.43 | 0.49 | 0.46 | 0.56 | 0.65 | 0.57 | 0.59 | 0.58 | 0.57 | 0.55 | 0.46 | 0.56 | 0.61 | 0.62 |
|  | **16-30** | 0.57 | 0.67 | 0.33 | 1.00 | 0.39 | 0.47 | 0.41 | 0.60 | 0.53 | 0.41 | 0.52 | 0.62 | 0.70 | 0.70 | 0.70 | 0.72 | 0.71 | 0.60 | 0.38 | 0.69 | 0.71 | 0.80 |
|  | **ANOVA** | n.s. | n.s. | n.s. | n.s. | <0.05 | n.s. | n.s. | n.s. | n.s. | n.s. | n.s. | n.s. | n.s. | n.s. | <0.05 | <0.01 | <0.05 | n.s. | n.s. | <0.05 | n.s. | <0.01 |
