# Supplementary material for: Tree Age Effects on Fine Root Biomass and Morphology over Chronosequences of Fagus sylvatica, Quercus robur and Alnus glutinosa Stands
Source: PLoS One. 2016 Feb 9;11(2):e0148668. doi: 10.1371/journal.pone.0148668 (PMC4747558; doi:10.1371/journal.pone.0148668)
Supplement: S4 Table — One-way ANOVAs were performed separately for the root traits studied to show significance of differences in fine root morphology between soil depths in each stand. Abbreviation: n.s. means not significantly different. (DOCX) [file pone.0148668.s007.docx]

**S4 Table**

| **Fine root traits** | **Soil depth**  **(cm)** | **Stand age (years)** | | | | | | | | | | | | | | | |
| --- | --- | --- | --- | --- | --- | --- | --- | --- | --- | --- | --- | --- | --- | --- | --- | --- | --- |
|  |  | **4** | **4** | **11** | **12** | **23** | **31** | **36** | **40** | **42** | **46** | **46** | **54** | **61** | **66** | **71** | **76** |
| **Diameter**  **(mm)** | **0-15** | 0.47 | 0.64 | 0.48 | 0.47 | 0.44 | 0.53 | 0.50 | 0.52 | 0.47 | 0.48 | 0.54 | 0.48 | 0.60 | 0.51 | 0.56 | 0.51 |
|  | **16-30** | 0.63 | 0.57 | 0.43 | 0.46 | 0.43 | 0.43 | 0.48 | 0.54 | 0.48 | 0.50 | 0.49 | 0.46 | 0.62 | 0.53 | 0.59 | 0.56 |
|  | **ANOVA** | n.s. | n.s. | n.s. | n.s. | n.s. | n.s. | n.s. | n.s. | n.s. | n.s. | n.s. | n.s. | n.s. | n.s. | n.s. | n.s. |
| **Length**  **(m m^-2^ soil)** | **0-15** | 770 | 285 | 1095 | 858 | 990 | 1315 | 1351 | 1044 | 783 | 658 | 2340 | 1830 | 790 | 1297 | 1827 | 634 |
|  | **16-30** | 154 | 181 | 346 | 163 | 608 | 803 | 536 | 474 | 898 | 242 | 835 | 507 | 293 | 437 | 1500 | 328 |
|  | **ANOVA** | <0.05 | n.s. | n.s. | <0.01 | n.s. | n.s. | <0.05 | n.s. | n.s. | <0.01 | <0.05 | <0.01 | n.s. | <0.05 | n.s. | n.s. |
| **Surface area**  **(m^2^ m^-2^ soil)** | **0-15** | 1.07 | 0.51 | 1.67 | 1.26 | 1.35 | 2.06 | 2.12 | 1.57 | 1.13 | 1.01 | 3.87 | 2.77 | 1.27 | 2.06 | 3.00 | 1.04 |
|  | **16-30** | 0.31 | 0.34 | 0.44 | 0.27 | 0.85 | 1.10 | 0.78 | 0.79 | 1.28 | 0.41 | 1.32 | 0.74 | 0.59 | 0.73 | 2.70 | 0.53 |
|  | **ANOVA** | <0.05 | n.s. | <0.05 | <0.01 | n.s. | n.s. | <0.01 | n.s. | n.s. | <0.01 | <0.05 | <0.01 | n.s. | <0.05 | n.s. | n.s. |
| **Volume**  **(m^3^ m^-2^ soil)** | **0-15** | 125 | 78 | 208 | 151 | 149 | 271 | 265 | 196 | 134 | 126 | 517 | 339 | 166 | 269 | 403 | 137 |
|  | **16-30** | 54 | 51 | 45 | 36 | 96 | 122 | 91 | 108 | 147 | 58 | 167 | 86 | 96 | 104 | 397 | 73 |
|  | **ANOVA** | n.s. | n.s. | <0.05 | <0.01 | n.s. | <0.05 | <0.01 | n.s. | n.s. | <0.05 | <0.05 | <0.01 | n.s. | n.s. | n.s. | n.s. |
| **No. of root tips**  **(×10^3^ m^-2^ soil)** | **0-15** | 183 | 67 | 267 | 196 | 244 | 259 | 275 | 244 | 189 | 153 | 460 | 426 | 167 | 283 | 391 | 121 |
|  | **16-30** | 36 | 43 | 93 | 37 | 179 | 196 | 127 | 118 | 208 | 56 | 179 | 134 | 71 | 104 | 333 | 79 |
|  | **ANOVA** | <0.05 | n.s. | n.s. | <0.01 | n.s. | n.s. | <0.05 | n.s. | n.s. | <0.01 | <0.05 | <0.01 | n.s. | <0.05 | n.s. | n.s. |
| **Root tip density**  **(tips m^-1^ fine roots)** | **0-15** | 220 | 227 | 250 | 234 | 246 | 207 | 208 | 238 | 242 | 237 | 203 | 234 | 267 | 216 | 208 | 212 |
|  | **16-30** | 247 | 318 | 279 | 259 | 306 | 232 | 232 | 248 | 231 | 244 | 235 | 264 | 290 | 261 | 215 | 263 |
|  | **ANOVA** | n.s. | n.s. | n.s. | n.s. | <0.01 | n.s. | <0.05 | n.s. | n.s. | n.s. | n.s. | <0.05 | n.s. | <0.05 | n.s. | <0.05 |
| **Specific root tip density**  **(tips g^-1^ fine roots)** | **0-15** | 3225 | 1931 | 3734 | 5070 | 3422 | 2124 | 2412 | 2702 | 3749 | 2302 | 2711 | 3208 | 2717 | 2511 | 1620 | 2824 |
|  | **16-30** | 3793 | 2235 | 6446 | 5210 | 4675 | 3243 | 3945 | 3047 | 3218 | 3482 | 5122 | 3844 | 3367 | 3606 | 1848 | 2402 |
|  | **ANOVA** | n.s. | n.s. | n.s. | n.s. | <0.05 | <0.05 | <0.05 | n.s. | n.s. | n.s. | n.s. | n.s. | n.s. | n.s. | n.s. | n.s. |
| **Specific root area**  **(cm^2^ g^-1^ fine roots)** | **0-15** | 214 | 149 | 213 | 311 | 187 | 158 | 184 | 181 | 227 | 143 | 227 | 204 | 184 | 178 | 136 | 205 |
|  | **16-30** | 239 | 121 | 324 | 243 | 202 | 191 | 249 | 209 | 203 | 200 | 347 | 207 | 197 | 200 | 151 | 153 |
|  | **ANOVA** | n.s. | n.s. | n.s. | n.s. | n.s. | n.s. | <0.05 | n.s. | n.s. | n.s. | n.s. | n.s. | n.s. | n.s. | n.s. | n.s. |
| **Specific root length**  **(m g^-1^ fine roots)** | **0-15** | 14.7 | 8.5 | 15.1 | 21.6 | 13.8 | 10.4 | 11.6 | 11.5 | 15.5 | 9.6 | 13.5 | 13.7 | 10.9 | 11.7 | 7.8 | 13.1 |
|  | **16-30** | 15.0 | 7.7 | 23.8 | 19.3 | 15.1 | 14.7 | 16.8 | 12.4 | 13.6 | 13.5 | 23.0 | 14.4 | 11.1 | 13.7 | 8.4 | 8.7 |
|  | **ANOVA** | n.s. | n.s. | n.s. | n.s. | n.s. | n.s. | <0.05 | n.s. | n.s. | n.s. | n.s. | n.s. | n.s. | n.s. | n.s. | n.s. |
| **Root tissue density**  **(g cm^-3^ fine roots)** | **0-15** | 0.52 | 0.47 | 0.43 | 0.43 | 0.51 | 0.79 | 0.45 | 0.83 | 0.42 | 0.60 | 0.52 | 0.42 | 0.51 | 0.63 | 0.56 | 0.43 |
|  | **16-30** | 0.33 | 0.60 | 0.41 | 0.43 | 0.49 | 0.54 | 0.36 | 0.39 | 0.45 | 0.86 | 0.52 | 0.43 | 0.38 | 0.65 | 0.47 | 1.08 |
|  | **ANOVA** | <0.05 | n.s. | n.s. | n.s. | n.s. | n.s. | n.s. | n.s. | n.s. | n.s. | n.s. | n.s. | n.s. | n.s. | n.s. | n.s. |
|  |  |  |  |  |  |  |  |  |  |  |  |  |  |  |  |  |  |
